# A new genus and a new species in the tribe Eryciini (Diptera, Tachinidae) from Area de Conservación Guanacaste in north-western Costa Rica

**DOI:** 10.3897/BDJ.13.e161853

**Published:** 2025-12-17

**Authors:** A.J. Fleming, M. Alex Smith, Winnie Hallwachs, Daniel Janzen

**Affiliations:** 1 Agriculture Agri-Food Canada, Ottawa, Canada Agriculture Agri-Food Canada Ottawa Canada; 2 University of Guelph, Guelph, Canada University of Guelph Guelph Canada; 3 Emeritus, Department of Biology, University of Pennsylvania, Philadelphia, Philadelphia, United States of America Emeritus, Department of Biology, University of Pennsylvania, Philadelphia Philadelphia United States of America

## Abstract

**Background:**

A new genus and species of within the tribe Eryciini are described from the Area de Conservación Guanacaste (ACG) in north-western Costa Rica. Specimens were reared from wild-caught caterpillars collected during an ongoing biodiversity inventory within the ACG. An integrative taxonomic approach was used to characterise the new taxa, incorporating morphological analysis, life history data and DNA barcodes, supported by high-resolution photographs. Furthermore, two new combinations are proposed and the associated species are re-described. A lectotype is designated for *Exorista loxostegae* (Reinhard, 1922) and an identification key to the species of the new genus is provided.

**New information:**

The description of the new genus *Santarosamyia* Fleming and Wood, 2024 gen. nov. along with the new species: *Santarosamyia woodorum* Fleming & Wood **sp. nov.** are provided.

The following new combinations are proposed: *Nilea erecta* (Coquillett, 1902) as *Santarosamyia erecta* (Coquillett, 1902) **comb. nov.**; *Nilea uniplilum* (Aldrich & Webber, 1924) as *Santarosamyia unipilum* (Aldrich & Webber, 1924) **comb. nov.**

A lectotype is designated for *Exorista loxostegae* (Reinhard, 1922).

Tachinidae, Diptera, Costa Rica, CO1, Parasitoid, Guanacaste

## Introduction

This paper describes a new genus, *Santarosamyia* **gen. nov.**, and one new species within the tribe Eryciini, reared from Lepidoptera larvae collected in the Area de Conservación Guanacaste (ACG), Costa Rica. The Eryciini is a diverse and incompletely defined tribe, first proposed by van Emden (1954) as an assemblage of genera within the subfamily Goniinae that he could not place elsewhere. The original diagnosis of the tribe included the presence of proclinate ocellar setae, two notopleural setae, three to four katepisternals and a row of black setulae posterior to the occipital row. The current concept of the tribe includes 128 separate genera in a polyphyletic group that continues to serve as a repository for exoristine taxa that cannot be placed satisfactorily into other tribes (Stireman 2002, Tachi and Shima 2010, Stireman et al. 2019).

The placement of genera within Eryciini is often challenging due to a lack of comprehensive revisions and overlapping morphological characters. Of particular relevance to this study is the genus *Nilea* Robineau-Desvoidy. In the absence of a modern revision, we follow Bergström (2007) in treating *Nilea* in the strict sense for comparative purposes. Although *Santarosamyia* shares some external characteristics with both *Nilea* and *Lespesia* Coquillett, making it difficult to distinguish by habitus alone, its status as a distinct genus is confirmed by an integrative analysis of the male postabdomen, DNA barcode data and external morphology. Based on this evidence, we also propose the transfer of two species formerly ascribed to *Nilea* into this new genus.

*Santarosamyia* possesses the general suite of characteristics or ‘gestalt’ used to place it within the tribe Eryciini and exhibits an affinity for caterpillars within the families Crambidae, Notodontidae, Noctuidae, Pyralidae and Tortricidae (Guimarães 1977, Arnaud 1978). However, we caution that additional rearing records are required to establish more robust conclusions regarding host range and specificity.

## Materials and methods

The rearing information and flies presented herein were collected by the ongoing ACG inventory of the caterpillars, their food plants and their parasitoids (Smith et al. 2006, Smith et al. 2007, Smith et al. 2008, Smith et al. 2009, Janzen et al. 2009, Janzen and Hallwachs 2011, Smith et al. 2012). The rearing methods used are described in detail at http://janzen.bio.upenn.edu/caterpillars/methodology/how/parasitoid_husbandry.htm.

This inventory has reared more than 750,000+ wild-caught caterpillars since its inception, collected throughout the major ACG terrestrial ecosystems (Janzen et al. 2009, Janzen and Hallwachs 2011, Janzen and Hallwachs 2020). This effort continues to provide an unprecedented amount of data, providing an invaluable tri-trophic database image of parasitoid biology, including parasitoids, their hosts and food plants. All frequencies of parasitisation reported here need to be considered against this background inventory, which will be analysed in detail in future works by DHJ, WH and co-authors.

The present work contributes to a series of publications documenting the tachinid fauna of the Area de Conservación Guanacaste (ACG) (http://www.acguanacaste.ac.cr), providing names to new species as they are described (Fleming et al. 2014a, Fleming et al. 2018, Fleming et al. 2019, Fleming et al. 2023). The primary goal of this series is to establish a taxonomic foundation for future, detailed studies of the ecology and behaviour of organisms and assemblages throughout ACG.

### Imaging and dissections

The species accounts and descriptions presented in this paper are deliberately concise and complemented with a series of colour photos used to illustrate their morphological differences and similarities. The morphological terminology used follows the most recent anatomical terminology outlined in the manual of Afrotropical Diptera by Cumming and Wood (2017). Descriptions are systematically arranged from anterior to posterior under the headings: **Head**, **Thorax**, **Abdomen** and **Male terminalia**. Although species can often be identified, based on external characters, the male postabdomen, which is characteristic for the genus, sometimes require dissection for positive identification. All procedures for dissection and photography followed the methods detailed by Fleming et al. (2014a). Measured landmarks of the head and examples of anatomical landmarks of the postabdomen discussed herein are illustrated in Figs 1, 2. Whenever possible, males were selected as holotypes as they typically bear the most distinct morphological characters for species recognition. In cases where only a single male specimen was available, it was designated the holotype and was not subjected to dissection.

### Voucher specimen management

The management of voucher specimens has been detailed in previous papers in this series (Fleming et al. 2014, Fleming et al. 2015, Fleming et al. 2018, Fleming et al. 2019, Fleming et al. 2020, Fleming et al. 2023). In brief, caterpillars reared by the ACG inventory receive a unique voucher code in the format yy–SRNP–xxxxx. Parasitoids emerging from a caterpillar receive the same voucher code; when/if they are later individually processed for DNA barcoding, each specimen receives a second, unique voucher code in the format DHJPARxxxxxxx. All associated data and successful barcodes are permanently and publicly deposited in the Barcode of Life Data System (BOLD) (Ratnasingham and Hebert 2007). All inventoried specimens discussed herein were collected under Costa Rican government research permits issued to DHJ and the Tachinidae samples were exported under permit by DHJ from Costa Rica to their final depository in the CNC. Tachinid identifications for the inventory are done by DHJ in coordination with: a) visual inspection of morphology by AJF and MW; b) DNA barcoding by MAS and BIO and c) databasing and association with host caterpillars by DHJ and WH through the inventory itself.

The date of capture cited for each specimen is the date of eclosion of the fly and not the date of capture of the host caterpillar. Eclosion date is much more representative of the time when that fly species is on the wing and, therefore, caught by net or Malaise trap than is the time of capture of the parasitised caterpillar. The “collector” is the parataxonomist who found the caterpillar rather than the person who later retrieved the newly-eclosed fly and processed it by freezing, pinning, labelling and oven-drying. The primary type material and additional specimens of the newly-described species are housed in the Diptera Collection of the Canadian National Collection (CNC).

### Acronyms for depositories

- CNC - Canadian National Collection of Insects, Arachnids and Nematodes, Ottawa, Canada
- USNM - National Museum of Natural History, Washington, D.C., U.S.A. (formerly United States National Museum)

### DNA Barcoding

DNA extraction from the legs of specimens was performed by first evaporating the ethanol from the plates and subsequently replacing it with 50 µl of tissue lysis buffer (1 M KCl, 20 mg/ml Proteinase K). The plates were then incubated overnight at 56°C to ensure complete lysis. Following lysis, DNA purification was achieved using SPRI beads (Rohland and Reich 2012). For samples prepared before 2018, a glass fibre protocol was used for DNA extraction (Ivanova et al. 2006).

The amplification of the COI barcode region was conducted via PCR, utilising a cocktail of Folmer primers (Folmer et al. 1994), along with LepF1 and LepR1 primers (Hebert et al. 2004). Most barcode amplicons were sequenced using Sanger technology; however, the most recently barcoded specimens were sequenced by SEQUEL II (Hebert et al. 2018).

The resulting DNA barcode sequences were then uploaded to the Barcode of Life Data System (BOLD) (Ratnasingham and Hebert 2007). BOLD can be consulted for metadata associated with each sequence by using the persistent DOI: https://doi.org/10.5883/DS-ASNILEA.

Barcode Index Numbers (or BINs) are assigned to high-quality sequences in BOLD automatically (Ratnasingham and Hebert 2007) and can be used as an initial species hypothesis. We used BINs as a species proxy to search for other species of Eryciini within reared tachinids of ACG with which to make genetic comparisons across genera within the tribe and for other publicly available *Nilea* sequences from North America.

### Interim names for undescribed species

Names of undescribed species follow a standardised interim system used for taxonomic units considered distinct species and identified by DNA barcodes. The interim names are given in the format *Nilea* Wood01 or tachinidWood12 Wood01, where the species epithet is either composed of the name of the taxonomist who identified the species and a number or the name of a morphospecies group, followed by a code fused with it. This prevents confusion with already-described species, while maintaining the traceability of each undescribed species within the ACG project.

## Taxon treatments

### Santarosamyia

Fleming & Wood gen. nov.

AE6DB8A0-7B1B-58EB-A993-B29B5DCA520A

D3F4D2B8-ADC4-4B0A-B1A8-7B32AB47CF23

***erecta*** Coquillett, 1902: 112 (*Phorocera*). Holotype female (USNM: USNMENT01789077) (examined by AJF). Type locality: USA, Arkansas, Camden. **comb. nov.**
loxostegeae Reinhard, 1922: 331 (*Exorista*). Syntypes, 14 males and 10 females (CNC) (examined by AJF). Type localities: USA, Texas, College Station.
***unipilum*** Aldrich & Webber, 1924: 83 (Phorocera (Neopales)). Holotype male (USNM: USNMENT01789003) (examined by AJF). Type locality: USA, Oregon, Hood River. Nearctic species. **comb. nov.**
***woodorum*** Fleming & Wood, 2025. Holotype male (CNC: DHJPAR0019111). Type locality: Costa Rica, Guanacaste, Area de Conservacíon Guanacaste, Sector Santa Rosa. **sp. nov.**
Santarosamyia woodorum Fleming & Wood, 2025. Type species.

#### Description

**Male**, **Head**: head slightly wider than thorax; vertex 1/4–1/3 head width; gena 1/6 of head height, approximately 1/5 of eye height; head width approximately 1/2 of height at widest point, giving it a slightly protruding character when viewed laterally, with one row of frontal setae, these extending well below base of pedicel and one pair of reclinate orbital setae, nearly in line with frontal row; frontal vitta at least as wide as either parafrontal, ocellar setae present and proclinate; eye densely setulose; parafacial bare and narrow; fronto-orbital plate ranging from shining silver to gold, with a sparse vestiture of short black irregularly inserted setulae; lower margin of face level with vibrissa; facial ridge with strong robust setae, along at least 1/3–2/3 its length almost reaching height of lowest frontal seta; pedicel black; postpedicel black, 6x as long as pedicel in males and 4x as long as pedicel in females; arista bare, usually distinctly-thickened on basal 4/5 almost to tip, palpus black to dark brown and setulose.

**Thorax**: scutum ranging from dark grey-back to brilliant gold; 4–5 dorsal vittae, these can often be bold and unbroken to only scarcely present under certain angles of light, outer pair broken at suture; prosternum setose; postpronotum bearing 4–5 setae, middle basal seta in line with outer and inner basal setae; anterior margin of anepisternum setulose with long hair-like black setulae. Thoracic chaetotaxy: acrostichal setae 3:3; dorsocentral setae 3:4; intra-alar setae 3:3; supra-alar setae 2:3; 3–4 katepisternal setae often widely spaced such that anterior katepisteral seta is closely paired with anterior ventral katepisternal seta; scutellum ground colour black with tomentosity ranging from nearly glabrous black to gold tomentose, with one pair of discal setae and three pairs of long flat marginal setae of subequal length; apical setae strong and crossed, inserted on the same plane as marginal setae, in some cases, at an upward angle from the plane of the rest of the scutellar marginal setae. **Legs**: black, with yellow pulvilli of varying length; hind coxae bare. Anterodorsal row of setae on hind tibia ciliate with up to 3–4 longer setae emerging above the rest. **Wing**: mostly pale translucent, to slightly hyaline; wing vein R_4+5_ setose, bearing only 1–3 setulae at base; calypters ranging from white to slightly yellow infuscate, almost amber coloured.

**Abdomen**: ground colour black dorsally with some orange laterally, tomentosity ranging from greyish–brown to nearly glabrous black; abdominal tomentosity ranging from strikingly gold to dull grey, often forming conspicuous anterior bands on dorsal surfaces of tergites, bisected by a narrow median black stripe; mid-dorsal depression on ST1+2 ranging from halfway across tergite to almost reaching to hind margin; one pair of median marginal setae present on ST1+2–T3 and a complete marginal row on T4 and T5; median discal setae present on T3–T5.

**Male terminalia**: sternite 5 with a deeply excavated median cleft along posterior edge, approximately 1.4x as wide as long, V-shaped, inner margins covered in dense tomentum; posterior lobes flattened somewhat apically, 2–3 strong setae surrounded by many shorter, weaker setulae; unsclerotised "window" on anterior plate of sternite 5 almost entirely translucent, shape ranging from rectangular to almost indistinct directly basal to posterior lobes. Epandrium setulose, cercus triangular, slightly longer than surstyli; cercus apically pointed, completely separate along most of its entire length. Cercus in lateral view, with a slight downward curve at apex, appearing slightly clubbed appearance to sharp and smoothly curved almost beak-like appearance, densely setulose along basal 2/3. Surstylus in lateral view, wide and robust, round medially, rounded and blunt at apex, not tapering to a point, giving the structure a wide digitate appearance; surstylus not fused with epandrium; when viewed dorsally, surstyli wide, slightly divergent, bearing a slight outward bend at apices. Pregonite broad, well-developed, apically rounded, blunt, with 6–7 setae along margin. Postgonite, slightly narrowed, up to 1/3 as wide as pregonite, sharply curved at apex. Basiphallus with a well-developed narrow elongate epiphallus; distiphallus broad with a thickened median process of the dorsal sclerite on its posterior surface pointed with a distinctive downward curve at apex and a broad, lateroventral sclerite, on the anterior surface also curving downwards at the apex.

**Female**, as in male, differing in the following traits: **Head**: bearing two pairs of proclinate orbital setae, frons wider than males. **Abdomen**: often slightly more globose than males. In those cases where sexual dimorphism is observed, the differing character states are mentioned in the species description.

#### Diagnosis

The genus *Santarosamyia* can be recognised by the combination of the following character states. Head, frons with a single row of frontal setae, parafacial bare, facial ridge strongly setose along lower 1/2–5/8, ocellar setae well developed and proclinate, eyes setulose, with distinct ommatrichia present and distance between eye and facial margin greater than 1/6 head height. Thorax, postpronotum with middle basal seta in a straight line, first postsutural supra-alar seta, as long and stout as first postsutural dorsocentral seta, 4 katepisternal setae, apical scutellar setae long and crossed often angled upwardly, but sometimes flat. Legs, hind coxa bare on postero-apical margin, tibia of last leg ciliate along posteroventral margin. It can be distinguished from *Lespesia* by the presence of both median discal and median marginal abdominal setae on tergites 2–4; it is distinguished from *Nilea* by its DNA barcode and the distinctive curvature at the apex of the median process of the dorsal sclerite, which is straight in both *Nilea* and *Lespesia* and presents as a distinctive character of the postabdomen in the new genus.

**Figure 3.**
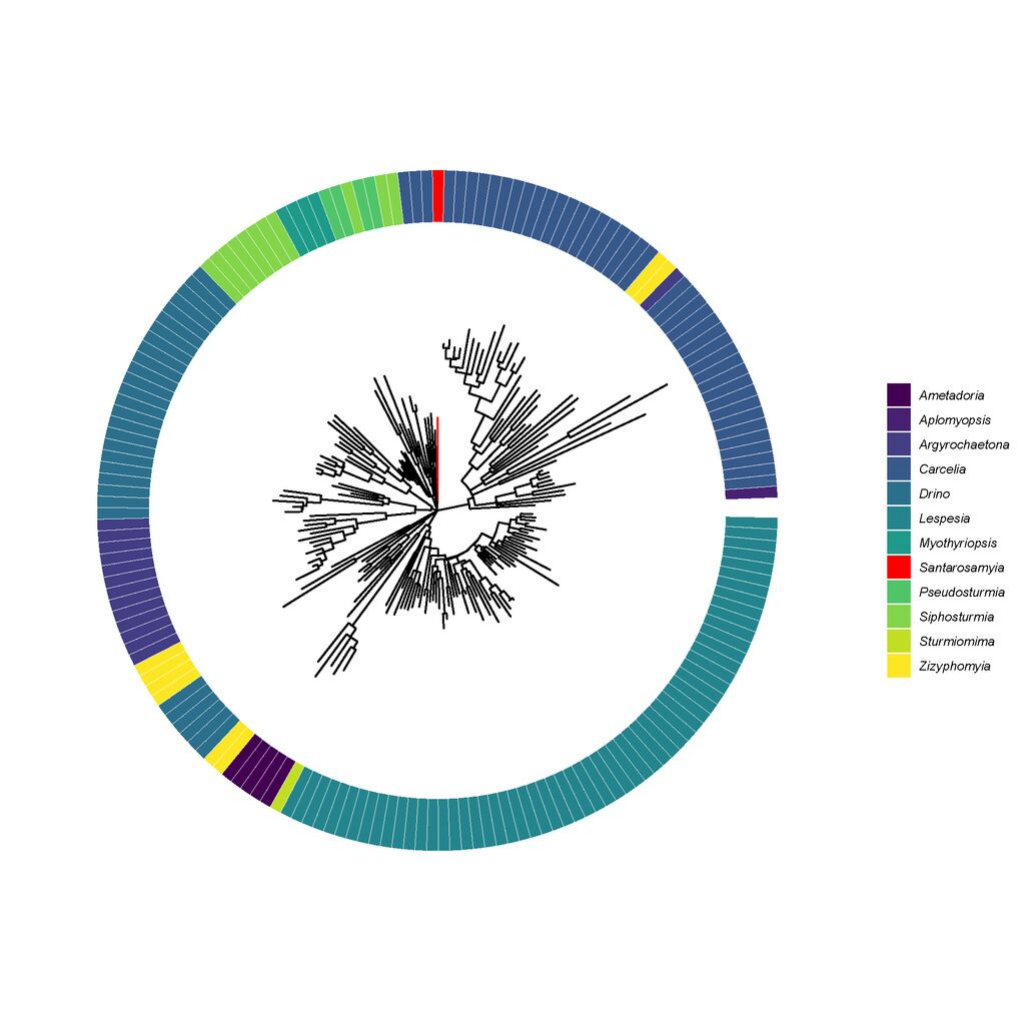
Phylogeny of 181 BINS from 12 genera of tachinid flies reared in ACG Costa Rica from the Eryciini. *Santarosamyia* **gen. nov.** shown in red.

The phylogeny of 181 BINS from 12 genera of tachinid flies reared in ACG Costa Rica from the Eryciini was estimated using the Maximum Likelihood method and General Time Reversible model (Nei and Kumar 2023) in MEGA11 (Tamura et al. 2021) (Fig. 3). *Santarosamyia* is, on average, 12% divergent from other genera within the ACG Eryciini and the average divergence between genera was 12%. Sequence divergence between genera was estimated using the Maximum Composite Likelihood model in MEGA11. Sample information is provided in Suppl. materials 1, 2.

**Figure 4.**
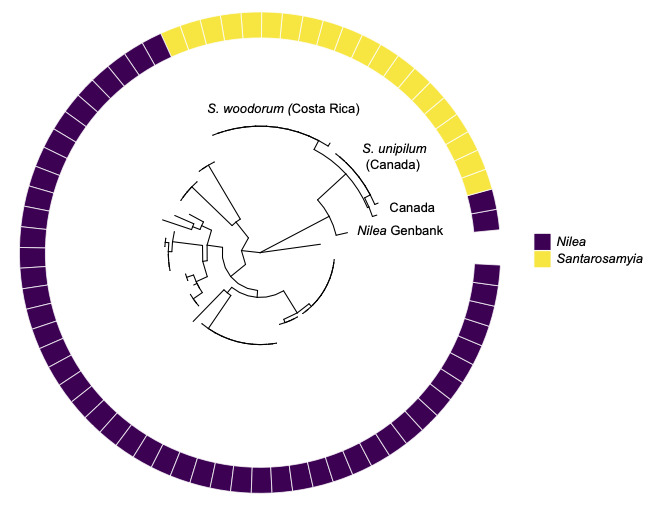
Phylogeny of 71 public sequences of *Santarosamyia* (*woodorum* and *unipilum*) and of public sequences of *Nilea. Santarosamyia* in yellow and public sequences of *Nilea* available on BOLD in purple.

A phylogeny of 71 public sequences of *Santarosamyia* (*woodorum* and *unipilum*) and of public sequences of *Nilea* was estimated using the Maximum Likelihood method and General Time Reversible model (Nei and Kumar 2023) in MEGA11 (Tamura et al. 2021) (Fig. 4). Our results suggest *Santarosamyia* is, on average, 10% divergent from *Nilea* and the average divergence within *Nilea* is 5%. Sequence divergence between genera was estimated using the Maximum Composite Likelihood model in MEGA1 (associated data provided in Suppl. material 3). Within Costa Rican *Santarosamyia* collected in ACG, there was only one specimen that posessed a single base-pair of variation in the DNA barcode region (an asynonymous A/G transition variation) and is 10% divergent from species of *Nilea*. *S. woodorum* DNA barcode sequences are AT rich, as is characteristic for insect mitochondrial DNA (AT content is 70.02%).

#### Etymology

The prefix of the compound name *Santarosamyia* refers to the locality where the holotype of the type species was collected (“Parque Nacional Santa Rosa” which is today Sector Santa Rosa of ACG). The suffix “myia” is in reference to the Greek word for fly.

#### Distribution

Widespread throught the Nearctic and Mesoamerican Regions, its range including Canada and USA, south to Costa Rica.

#### Ecology

To date, *Santarosamyia* has been reared from Lepidoptera larvae in the families: Crambidae, Geometridae, Olethreutidae and Pyralidae.

#### Taxon discussion

*Santarosamyia* keys out in the Manual of Nearctic Diptera (Wood 1987) with couplets 43 and 46 along with *Nilea*. However, *Santarosamyia* does not consistently possess horizontally directed apical scutellar setae, nor the diagnostic placement of the katepisternal setae, both features used to distinguish *Nilea* from *Lespesia*. In the Manual of Central American Diptera (Wood and Zumbado 2010), it keys out with *Lespesia* in couplet 57. It is distinguished from these closely-related genera by the presence of discal setae on tergites 3 and 4.

### Santarosamyia woodorum

Fleming & Wood sp. nov.

A01E1D8F-7526-5DF8-B127-66F7FD7493A2

71FA7D1B-3CAC-45CE-9781-7B85AF86D186

#### Materials

- **Type status:** Holotype. **Occurrence:** occurrenceDetails: http://janzen.sas.upenn.edu; catalogNumber: DHJPAR0019111; recordedBy: D.H. Janzen, W. Hallwachs & gusaneros; individualID: DHJPAR0019111; individualCount: 1; sex: M; lifeStage: adult; preparations: pinned; otherCatalogNumbers: 00-SRNP-18594, BOLD:AAA1961, ASTAI1758-07; occurrenceID: 8CF487EE-BF53-59E4-A5BB-6F042019AC6D; **Taxon:** scientificName: *Santarosamyia woodorum*; phylum: Arthropoda; class: Insecta; order: Diptera; family: Tachinidae; genus: Santarosamyia; specificEpithet: *woodorum*; scientificNameAuthorship: Fleming & Wood, 2025; **Location:** continent: Central America; country: Costa Rica; countryCode: CR; stateProvince: Guanacaste; county: Sector Santa Rosa; locality: Area de Conservacion Guanacaste; verbatimElevation: 295; verbatimLatitude: 10.837600; verbatimLongitude: -85.618700; verbatimCoordinateSystem: Decimal; decimalLatitude: 10.8376; decimalLongitude: -85.6187; **Identification:** identifiedBy: AJ Fleming; dateIdentified: 2024; **Event:** samplingProtocol: Reared from a Crambidae larva, Eulepte Janzen06 00-SRNP-18594; verbatimEventDate: 24-Oct-2000; **Record Level:** language: en; institutionCode: CNC; collectionCode: Insects; basisOfRecord: Pinned Specimen
- **Type status:** Paratype. **Occurrence:** occurrenceDetails: http://janzen.sas.upenn.edu; catalogNumber: DHJPAR0019109; recordedBy: D.H. Janzen, W. Hallwachs & gusaneros; individualID: DHJPAR0019109; individualCount: 1; sex: M; lifeStage: adult; preparations: pinned, dissected; otherCatalogNumbers: 00-SRNP-18614, BOLD:AAA1961, ASTAI1756-07; occurrenceID: EF600D3A-022F-5E82-BDF1-0925F3CFFD05; **Taxon:** scientificName: *Santarosamyia woodorum*; phylum: Arthropoda; class: Insecta; order: Diptera; family: Tachinidae; genus: Santarosamyia; specificEpithet: *woodorum*; scientificNameAuthorship: Fleming & Wood, 2025; **Location:** continent: Central America; country: Costa Rica; countryCode: CR; stateProvince: Guanacaste; county: Sector Santa Rosa; locality: Area de Conservacion Guanacaste; verbatimLocality: Vado Nisperal; verbatimElevation: 295; verbatimLatitude: 10.837600; verbatimLongitude: -85.618700; verbatimCoordinateSystem: Decimal; decimalLatitude: 10.8376; decimalLongitude: -85.6187; **Identification:** identifiedBy: AJ Fleming; dateIdentified: 2024; **Event:** samplingProtocol: Reared from a Crambidae larva, Eulepte concordalis; verbatimEventDate: 19-Sep-2000; **Record Level:** language: en; institutionCode: CNC; collectionCode: Insects; basisOfRecord: Pinned Specimen
- **Type status:** Paratype. **Occurrence:** occurrenceDetails: http://janzen.sas.upenn.edu; catalogNumber: DHJPAR0019110; recordedBy: D.H. Janzen, W. Hallwachs & gusaneros; individualID: DHJPAR0019110; individualCount: 1; sex: M; lifeStage: adult; preparations: pinned; otherCatalogNumbers: 00-SRNP-18593, BOLD:AAA1961, ASTAI1757-07; occurrenceID: FA189C84-7C68-5E13-9009-52420C1E65ED; **Taxon:** scientificName: *Santarosamyia woodorum*; phylum: Arthropoda; class: Insecta; order: Diptera; family: Tachinidae; genus: Santarosamyia; specificEpithet: *woodorum*; scientificNameAuthorship: Fleming & Wood, 2025; **Location:** continent: Central America; country: Costa Rica; countryCode: CR; stateProvince: Guanacaste; county: Sector Santa Rosa; locality: Area de Conservacion Guanacaste; verbatimElevation: 295; verbatimLatitude: 10.837600; verbatimLongitude: -85.618700; verbatimCoordinateSystem: Decimal; decimalLatitude: 10.8376; decimalLongitude: -85.6187; **Identification:** identifiedBy: AJ Fleming; dateIdentified: 2024; **Event:** samplingProtocol: Reared from a Crambidae larva, Eulepte concordalis; verbatimEventDate: 22-Oct-2000; **Record Level:** language: en; institutionCode: CNC; collectionCode: Insects; basisOfRecord: Pinned Specimen
- **Type status:** Paratype. **Occurrence:** occurrenceDetails: http://janzen.sas.upenn.edu; catalogNumber: DHJPAR0017098; recordedBy: D.H. Janzen, W. Hallwachs & Lucia Vargas; individualID: DHJPAR0017098; individualCount: 1; sex: F; lifeStage: adult; preparations: pinned; otherCatalogNumbers: 07-SRNP-12386, BOLD:AAA1961, ASTAP536-07; occurrenceID: 72A34A57-D486-5A08-95E3-40839717FEC9; **Taxon:** scientificName: *Santarosamyia woodorum*; phylum: Arthropoda; class: Insecta; order: Diptera; family: Tachinidae; genus: Santarosamyia; specificEpithet: *woodorum*; scientificNameAuthorship: Fleming & Wood, 2025; **Location:** continent: Central America; country: Costa Rica; countryCode: CR; stateProvince: Guanacaste; county: Sector Santa Elena; locality: Area de Conservacion Guanacaste; verbatimLocality: Vado Nisperal; verbatimElevation: 20; verbatimLatitude: 10.847100; verbatimLongitude: -85.771400; verbatimCoordinateSystem: Decimal; decimalLatitude: 10.8471; decimalLongitude: -85.7714; **Identification:** identifiedBy: AJ Fleming; dateIdentified: 2024; **Event:** samplingProtocol: Reared from a Crambidae larva, Omiodes cuniculalis; verbatimEventDate: 27-Mar-2007; **Record Level:** language: en; institutionCode: CNC; collectionCode: Insects; basisOfRecord: Pinned Specimen
- **Type status:** Paratype. **Occurrence:** occurrenceDetails: http://janzen.sas.upenn.edu; catalogNumber: DHJPAR0019112; recordedBy: D.H. Janzen, W. Hallwachs & gusaneros; individualID: DHJPAR0019112; individualCount: 1; sex: F; lifeStage: adult; preparations: pinned; otherCatalogNumbers: 00-SRNP-18220, BOLD:AAA1961, ASTAI1759-07; occurrenceID: 33F7A385-7246-5057-B75B-68F67ABD95D7; **Taxon:** scientificName: *Santarosamyia woodorum*; phylum: Arthropoda; class: Insecta; order: Diptera; family: Tachinidae; genus: Santarosamyia; specificEpithet: *woodorum*; scientificNameAuthorship: Fleming & Wood, 2025; **Location:** continent: Central America; country: Costa Rica; countryCode: CR; stateProvince: Guanacaste; county: Sector Santa Elena; locality: Area de Conservacion Guanacaste; verbatimElevation: 290; verbatimLatitude: 10.863200; verbatimLongitude: -85.663200; verbatimCoordinateSystem: Decimal; decimalLatitude: 10.8632; decimalLongitude: -85.6632; **Identification:** identifiedBy: AJ Fleming; dateIdentified: 2024; **Event:** samplingProtocol: Reared from a Pyralidae larva, Chloropaschia granitalis; verbatimEventDate: 21-Oct-2000; **Record Level:** language: en; institutionCode: CNC; collectionCode: Insects; basisOfRecord: Pinned Specimen
- **Type status:** Paratype. **Occurrence:** occurrenceDetails: http://janzen.sas.upenn.edu; catalogNumber: DHJPAR0019797; recordedBy: D.H. Janzen, W. Hallwachs & Lucia Vargas; individualID: DHJPAR0019797; individualCount: 1; sex: M; lifeStage: adult; preparations: pinned; otherCatalogNumbers: 07-SRNP-12395, BOLD:AAA1961, ASTAB345-07; occurrenceID: 95D52D36-5C88-5C2C-B9FC-19C9752A6DE0; **Taxon:** scientificName: *Santarosamyia woodorum*; phylum: Arthropoda; class: Insecta; order: Diptera; family: Tachinidae; genus: Santarosamyia; specificEpithet: *woodorum*; scientificNameAuthorship: Fleming & Wood, 2025; **Location:** continent: Central America; country: Costa Rica; countryCode: CR; stateProvince: Guanacaste; county: Sector Santa Elena; locality: Area de Conservacion Guanacaste; verbatimElevation: 20; verbatimLatitude: 10.847100; verbatimLongitude: -85.771400; verbatimCoordinateSystem: Decimal; decimalLatitude: 10.8471; decimalLongitude: -85.7714; **Identification:** identifiedBy: AJ Fleming; dateIdentified: 2024; **Event:** samplingProtocol: Reared from a Crambidae larva, Omiodes cuniculalis; verbatimEventDate: 28-Mar-2007; **Record Level:** language: en; institutionCode: CNC; collectionCode: Insects; basisOfRecord: Pinned Specimen
- **Type status:** Paratype. **Occurrence:** occurrenceDetails: http://janzen.sas.upenn.edu; catalogNumber: DHJPAR0037222; recordedBy: D.H. Janzen, W. Hallwachs & Lucia Vargas; individualID: DHJPAR0037222; individualCount: 1; lifeStage: adult; preparations: pinned; otherCatalogNumbers: 07-SRNP-12403, BOLD:AAA1961, ASHYE2055-10; occurrenceID: 6F277DFA-0504-5494-89D3-5D4D3D1B230C; **Taxon:** scientificName: *Santarosamyia woodorum*; phylum: Arthropoda; class: Insecta; order: Diptera; family: Tachinidae; genus: Santarosamyia; specificEpithet: *woodorum*; scientificNameAuthorship: Fleming & Wood, 2025; **Location:** continent: Central America; country: Costa Rica; countryCode: CR; stateProvince: Guanacaste; county: Sector Santa Elena; locality: Area de Conservacion Guanacaste; verbatimElevation: 20; verbatimLatitude: 10.847100; verbatimLongitude: -85.771400; verbatimCoordinateSystem: Decimal; decimalLatitude: 10.8471; decimalLongitude: -85.7714; **Identification:** identifiedBy: AJ Fleming; dateIdentified: 2024; **Event:** samplingProtocol: Reared from a Crambidae larva, Omiodes cuniculalis; verbatimEventDate: 15-May-2007; **Record Level:** language: en; institutionCode: CNC; collectionCode: Insects; basisOfRecord: Pinned Specimen
- **Type status:** Paratype. **Occurrence:** occurrenceDetails: http://janzen.sas.upenn.edu; catalogNumber: DHJPAR0070383; recordedBy: D.H. Janzen, W. Hallwachs & Minor Carmona; individualID: DHJPAR0070383; individualCount: 1; sex: M; lifeStage: adult; preparations: pinned; otherCatalogNumbers: 22-SRNP-26507, BOLD:AAA1961, ACGBA15868-23; occurrenceID: E69D8967-3C76-5544-8AD6-0A00B7DC8393; **Taxon:** scientificName: *Santarosamyia woodorum*; phylum: Arthropoda; class: Insecta; order: Diptera; family: Tachinidae; genus: Santarosamyia; specificEpithet: *woodorum*; scientificNameAuthorship: Fleming & Wood, 2025; **Location:** continent: Central America; country: Costa Rica; countryCode: CR; stateProvince: Guanacaste; county: Sector Rincon Rain Forest; locality: Area de Conservacion Guanacaste; verbatimElevation: 326; verbatimLatitude: 10.970700; verbatimLongitude: -85.314300; verbatimCoordinateSystem: Decimal; decimalLatitude: 10.9707; decimalLongitude: -85.3143; **Identification:** identifiedBy: AJ Fleming; dateIdentified: 2024; **Event:** samplingProtocol: Reared from a Crambidae larva, Eulepte concordalis; verbatimEventDate: 04-Mar-2022; **Record Level:** language: en; institutionCode: CNC; collectionCode: Insects; basisOfRecord: Pinned Specimen
- **Type status:** Paratype. **Occurrence:** occurrenceDetails: http://janzen.sas.upenn.edu; catalogNumber: DHJPAR0070378; recordedBy: D.H. Janzen, W. Hallwachs & Minor Carmona; individualID: DHJPAR0070378; individualCount: 1; sex: F; lifeStage: adult; preparations: pinned; otherCatalogNumbers: 22-SRNP-26509, BOLD:AAA1961, ACGBA15869-23; occurrenceID: 1EFC71D9-58DB-54FD-AAE1-DE446454B19F; **Taxon:** scientificName: *Santarosamyia woodorum*; phylum: Arthropoda; class: Insecta; order: Diptera; family: Tachinidae; genus: Santarosamyia; specificEpithet: *woodorum*; scientificNameAuthorship: Fleming & Wood, 2025; **Location:** continent: Central America; country: Costa Rica; countryCode: CR; stateProvince: Guanacaste; county: Sector Rincon Rain Forest; locality: Area de Conservacion Guanacaste; verbatimElevation: 326; verbatimLatitude: 10.970700; verbatimLongitude: -85.314300; verbatimCoordinateSystem: Decimal; decimalLatitude: 10.9707; decimalLongitude: -85.3143; **Identification:** identifiedBy: AJ Fleming; dateIdentified: 2024; **Event:** samplingProtocol: Reared from a Crambidae larva, Eulepte concordalis; verbatimEventDate: 04-Mar-2022; **Record Level:** language: en; institutionCode: CNC; collectionCode: Insects; basisOfRecord: Pinned Specimen
- **Type status:** Paratype. **Occurrence:** occurrenceDetails: http://janzen.sas.upenn.edu; catalogNumber: DHJPAR0070379; recordedBy: D.H. Janzen, W. Hallwachs & Minor Carmona; individualID: DHJPAR0070379; individualCount: 1; sex: F; lifeStage: adult; preparations: pinned; otherCatalogNumbers: 22-SRNP-26512, BOLD:AAA1961, ACGBA15870-23; occurrenceID: 9BD0A312-5B2D-5CE2-AF21-89561BF43F58; **Taxon:** scientificName: *Santarosamyia woodorum*; phylum: Arthropoda; class: Insecta; order: Diptera; family: Tachinidae; genus: Santarosamyia; specificEpithet: *woodorum*; scientificNameAuthorship: Fleming & Wood, 2025; **Location:** continent: Central America; country: Costa Rica; countryCode: CR; stateProvince: Guanacaste; county: Sector Rincon Rain Forest; locality: Area de Conservacion Guanacaste; verbatimElevation: 326; verbatimLatitude: 10.970700; verbatimLongitude: -85.314300; verbatimCoordinateSystem: Decimal; decimalLatitude: 10.9707; decimalLongitude: -85.3143; **Identification:** identifiedBy: AJ Fleming; dateIdentified: 2024; **Event:** samplingProtocol: Reared from a Crambidae larva, Eulepte concordalis; verbatimEventDate: 04-Mar-2022; **Record Level:** language: en; institutionCode: CNC; collectionCode: Insects; basisOfRecord: Pinned Specimen
- **Type status:** Paratype. **Occurrence:** occurrenceDetails: http://janzen.sas.upenn.edu; catalogNumber: DHJPAR0070380; recordedBy: D.H. Janzen, W. Hallwachs & Minor Carmona; individualID: DHJPAR0070380; individualCount: 1; sex: F; lifeStage: adult; preparations: pinned; otherCatalogNumbers: 22-SRNP-26513, BOLD:AAA1961, ACGBA15871-23; occurrenceID: 682AEB94-D846-5F82-AFCB-1B0A7EC01D55; **Taxon:** scientificName: *Santarosamyia woodorum*; phylum: Arthropoda; class: Insecta; order: Diptera; family: Tachinidae; genus: Santarosamyia; specificEpithet: *woodorum*; scientificNameAuthorship: Fleming & Wood, 2025; **Location:** continent: Central America; country: Costa Rica; countryCode: CR; stateProvince: Guanacaste; county: Sector Rincon Rain Forest; locality: Area de Conservacion Guanacaste; verbatimElevation: 326; verbatimLatitude: 10.970700; verbatimLongitude: -85.314300; verbatimCoordinateSystem: Decimal; decimalLatitude: 10.9707; decimalLongitude: -85.3143; **Identification:** identifiedBy: AJ Fleming; dateIdentified: 2024; **Event:** samplingProtocol: Reared from a Crambidae larva, Eulepte concordalis; verbatimEventDate: 04-Mar-2022; **Record Level:** language: en; institutionCode: CNC; collectionCode: Insects; basisOfRecord: Pinned Specimen
- **Type status:** Paratype. **Occurrence:** occurrenceDetails: http://janzen.sas.upenn.edu; catalogNumber: DHJPAR0111696; recordedBy: D.H. Janzen, W. Hallwachs & Janzen2023; individualID: DHJPAR0111696; individualCount: 1; lifeStage: adult; preparations: pinned; otherCatalogNumbers: 23-SRNP-26255, BOLD:AAA1961, ACGBA16794-23; occurrenceID: 57EFF960-31A6-5B9E-9C5A-A9BD8ED4BA99; **Taxon:** scientificName: *Santarosamyia woodorum*; phylum: Arthropoda; class: Insecta; order: Diptera; family: Tachinidae; genus: Santarosamyia; specificEpithet: *woodorum*; scientificNameAuthorship: Fleming & Wood, 2025; **Location:** continent: Central America; country: Costa Rica; countryCode: CR; stateProvince: Guanacaste; county: Sector Rincon Rain Forest; locality: Area de Conservacion Guanacaste; verbatimCoordinateSystem: Decimal; **Identification:** identifiedBy: AJ Fleming; dateIdentified: 2024; **Record Level:** language: en; institutionCode: CNC; collectionCode: Insects; basisOfRecord: Pinned Specimen

#### Description

**Male** (Fig. 5), **Head**: vertex 1/3 head width; gena 1/6 of head height, approximately 1/5 of eye height; with one row of frontal setae, these extending below base of pedicel and one pair of reclinate orbital setae, nearly in line with frontal row; gena dark grey, covered in short black setulae; fronto-orbital plate dark grey with a silver sheen, covered in black setulae surrounding frontal setae; facial ridge setose, along lower 2/3 of its length; pedicel black; postpedicel black, 4x as long as pedicel; arista, distinctly thickened on the basal half. Palpus dark brown, almost black, densely setulose, digitiform, not distinctly clubbed.

**Thorax**: scutum black ground colour, covered in a grey tomentum which appears to fade along posterior edge; four dorsal vittae, outer pair broken at suture, extending beyond third postsutural dorsocentral seta, inner pair unbroken extending beyond 2^nd^ postsutural dorsocentral, vittae becoming more prominent under certain angles of light; postpronotum bearing four setae, middle basal seta in line with outer and inner basal setae; anterior margin of anepimeron with only 2–4 long setae. Chaetotaxy: acrostichal setae 3:3; dorsocentral setae 3:4; intra-alar setae 3:3; supra-alar setae 2:3; 4 katepisternal setae; scutellum black with dark maroon along basal edges, with one pair of discal setae and three pairs of long flat marginal setae; apical setae long.

**Abdomen**: ground colour reddish–brown laterally to black dorsally; abdominal tomentum dull gold, forming conspicuous bands on dorsal surfaces of T3–T5, these bands being bisected by a narrow median black stripe; median discal setae present T3 and T4.

**Male terminalia** (Fig. 6): sternite 5 with a deeply excavated median cleft along the posterior edge, approximately 1.4x as wide as long, V-shaped, inner margins covered in dense tomentum; posterior lobes flattened somewhat apically, two strong setae surrounded by many shorter, weaker setulae; unsclerotised "window" on anterior plate of sternite 5 almost entirely translucent, almost indistinct directly basal to posterior lobes. Epandrium setulose, cercus triangular, slightly longer than surstyli; cercus apically pointed, completely separate along most of their length. Cercus in lateral view, with a slight downward curve at apex, densely setose along basal 2/3. Surstylus in lateral view, wide and robust, round medially, rounded and blunt at apex, not tapering to a point, giving the structure a wide digitate appearance; surstylus not fused with epandrium; when viewed dorsally, surstyli wide, slightly divergent, bearing a slight outward bend at apices. Pregonite broad, well-developed, apically rounded, blunt, with 6–7 setae along margin. Postgonite, slightly narrowed, up to 1/2 as wide as pregonite, curved at apex. Basiphallus with a well-developed narrow and curved epiphallus, distiphallus broad with a thick median longitudinal sclerotised reinforcement on its posterior surface pointed with a distinctive downward curve at apex and a broad, anterolateral, sclerotised acrophallus, on the anterior surface also curving downwards at the apex.

**Female** (Fig. 7), as in male, differing in the following traits: **Head**: bearing two pairs of proclinate orbital setae and two pairs of reclinate orbital setae, palpus dark ochraceous appearing brown to black, antennal pedicel dark orange almost brown, but distinctly lighter than postpedicel. **Thorax**: tomentum more brilliant yellow–gold than male, thinner post suturally, but apparently extending over the entirety of thorax and scutellum. Wings slightly more hyaline than male. **Abdomen**: abdominal tomentum gold, overall appearing more globose than males and in its terminalia.

#### Diagnosis

*Santarosamyia woodorum* sp. nov. can be distinguished from other *Santarosamyia* by the following combination of traits: fronto-orbital plate dark grey with a silver sheen, covered in black setulae surrounding frontal setae, frontal setae extending below base of pedicel; facial ridge strongly setose along 2/3–4/5 of its length. *Santarosamyia woodorum* sp. nov. is separated in the key from *S. erecta* comb. nov. by the gold tomentosity of the thorax and abdomen, its pale white translucent lower calypters and by its COI sequence clustered within the Barcode Identification Number (BIN) BOLD:AAA1961.

The BOLD BIN BOLD:AAA1961 contains both *Santarosamyia woodorum* (from Costa Rica) and *S. unipilum* from Canada. The two species are easily differentiated in the barcode region by 8 bp (Fig. 3).

Consensus DNA barcode for *S. woodorum*:

ACTTTATATTTTATTTTTGGAGCCTGAGCTAGTATAATTGGAACATCTTTAAGTATATTAATTCGAATTGAATTAGGACATCCCGGTTCATTAATTGGAAATGATCAAATTTACAATGTAATTGTAACAGCTCATGCATTTGTTATAATTTTTTTTATAGTAATACCAATTATAATTGGAGGATTTGGTAATTGATTAGTTCCTTTAAT**R**TTAGGAGCCCCAGATATAGCCTTTCCACGAATAAATAATATAAGTTTTTGACTCCTTCCTCCTGCATTAACACTTTTATTAACAAGAAGTATAGTAGAAAGCGGATCTGGGACAGGATGAACAGTTTATCCCCCTTTATCTTCTATTATTGCTCATGGAGGAGCTTCTGTTGATTTAGCTATTTTTTCTTTACACTTAGCTGGAATTTCTTCTATTTTAGGAGCTGTAAATTTTATTACTACTGTTATTAATATACGATCATCAGGAATTACTTTTGATCGAATACCTTTATTTGTTTGATCAGTTGTTATTACAGCTTTATTACTCTTATTATCTTTACCTGTATTAGCCGGAGCTATTACTATATTATTAACAGATCGAAATTTAAATACATCATTTTTTGATCCAGCGGGAGGAGGTGATCCTATTTTATATCAACATTTATTT

#### Etymology

*Santarosamyia woodorum* sp. nov. is named in honour of Dr. D. Monty Wood (1933–2020) and Grace Wood for their many years of dedication to the study of Diptera and, in particular, the family Tachinidae of ACG. Their legacy lives in Monty's invaluable contributions to science and the countless people he educated and collaborated with. Interim species-specific names for *Santarosamyia woodorum* sp. nov., included in previously circulating databases and publications, are tachinidWood12 Wood01 and *Nilea* Wood01.

#### Distribution

Costa Rica, ACG, Guanacaste Province, 20–326 m elevation.

#### Ecology

*Santarosamyia woodorum* sp. nov. has been reared twelve times from at least two species of Lepidoptera in the family Crambidae: *Omiodes cuniculalis* Guenée, 1854 (n = 3), *Eulepte* Janzen06 (n = 1) and *Eulepte concordalis* Hübner, 1825 (n = 7) and one species in the family Pyralidae: *Chloropaschia granitalis* (C. Felder, R. Felder & Rogenhofer, 1875) (n = 1) in dry forest, at elevations ranging from 20–326 m.

### Santarosamyia erecta

(Coquillett, 1902) comb. nov.

FB4577E4-0C43-59A1-BE57-67978309A911

erecta Coquillett, 1902: 112 (*Phorocera*). Holotype female by designation of Coquillett, 1902: 112 (USNM: USNMENT01789077) (Examined by: AJF and DMW). Type locality: USA, California, Camden Ark. **comb. nov.**
loxostegeae Reinhard, 1922: 331 (*Exorista*). Lectotype male by present designation (CNC:CNC1175758). Type locality: USA, Texas, College Station.

#### Materials

- **Type status:** Lectotype. **Occurrence:** recordedBy: H.J. Reinhard; occurrenceID: 0F8D89CC-56C4-5E2B-95E0-6D8ED2D4BD99; **Taxon:** scientificNameID: Exorista loxostegae; namePublishedInID: Reinhard, 1921; scientificName: *Exorista loxostegae* Reinhard, 1921; nameAccordingTo: Reinhard, 1921; order: Diptera; family: Tachinidae; genus: Exorista; specificEpithet: loxostegae; taxonRemarks: Lectotype of Exorista loxostegae (Reinhard, 1922) by present designation of Fleming & Wood; **Location:** continent: North America; country: United States of America; countryCode: US; stateProvince: Texas; locality: College Station; verbatimLocality: College Station, Texas; **Identification:** identifiedBy: Reinhard; dateIdentified: 1921; **Event:** year: 20; month: 6; day: 14; verbatimEventDate: 6-14-20; **Record Level:** type: pinned adult specimen; collectionID: CNC1175758; institutionCode: CNC; basisOfRecord: Preserved specimen
- **Type status:** Other material. **Occurrence:** recordedBy: J.I. Beauine; sex: M; preparations: terminalia dissected; occurrenceID: 5BA60566-D9B7-501C-8994-0036E842D866; **Taxon:** scientificName: *Nilea erecta* (Coquillett, 1902); kingdom: Animalia; class: Insecta; order: Diptera; family: Tachinidae; genus: Nilea; specificEpithet: erecta; **Location:** continent: North America; country: Canada; countryCode: CA; stateProvince: Quebec; municipality: Hull; verbatimLocality: Hull, P.Q.; **Identification:** identifiedBy: H.J. Reinhard; **Event:** eventDate: 18 July 1914; year: 1914; month: July; day: 18; verbatimEventDate: 18.VII.1914; eventRemarks: Compare with the type in USNM; **Record Level:** institutionID: CNC; institutionCode: CNC

#### Description

**Male** (Fig. 8), **Head**: head slightly wider than thorax when viewed dorsally; vertex 1/3 head width; gena 1/6 of head height, approximately 1/5 of eye height; with one row of frontal setae, these extending below base of pedicel and two pairs of reclinate orbital setae, nearly in line with frontal row; ocellar setae strong and proclinate, inserted directly adjacent to anterior ocellus; eye setulose; parafacial bare and narrow, slightly grey tomentose; gena grey, covered in short black setulae; fronto-orbital plate grey tomentose, covered in black setulae surrounding frontal setae; lower margin of face level with vibrissa; facial ridge setose, along most of its length, almost reaching lowest frontal seta; pedicel black; postpedicel black, 4x as long as pedicel; arista bare, distinctly-thickened on basal half. Palpus dark brown, almost black, densely setulose, digitiform, not distinctly clubbed.

**Thorax**: scutum black ground colour, grey tomentose; four dorsal vittae, almost indistinct, outer pair broken at suture, vittae becoming more prominent under certain angles of light; postpronotum bearing four setae, middle basal seta in line with outer and inner basal setae; anterior margin of anipemeron with only 2–4 long setae. Chaetotaxy: acrostichal setae 3:3; dorsocentral setae 3:4; intra-alar setae 3:3; supra-alar setae 2:3; 3 katepisternal setae; scutellum black with dark maroon along basal edges, with one pair of discal setae and three pairs of long flat marginal setae.

**Abdomen**: ground colour reddish-brown to black (original description states black, discrepancy could be due to the age of the specimens examined, lightening as they age); tergites 3–4 thinly grey tomentose, extending almost to the apex of the tergites; median discal setae present T3 and T4.

**Male terminalia** (Fig. 9): sternite 5 with a deeply excavated median cleft along posterior edge, approximately 1.4x as wide as long, U-shaped, inner margins covered in light tomentum; posterior lobes flattened somewhat apically, two strong setae surrounded by many shorter, weaker setulae; unsclerotised "window" on anterior plate of sternite 5 almost entirely translucent, directly basal to posterior lobes. Epandrium setulose, cercus triangular, slightly longer than surstyli; cercus apically pointed, completely separate along most of their length. Cercus in lateral view, with a slight downward curve at apex, densely setulose along basal 2/3. Surstylus in lateral view, wide and robust, round medially, rounded and blunt at apex, slightly tapered basally, giving the structure a wide digitate to slightly spatulate appearance; surstylus not fused with epandrium; when viewed dorsally surstyli wide, slightly divergent, bearing a slight outward bend at apices. Pregonite broad, well-developed, apically rounded, blunt, with 6–7 setae along margin. Postgonite, up to 1/2 as wide as pregonite, curved apex. Basiphallus with a well-developed narrow and erect epiphallus, distiphallus broad with a thick median longitudinal sclerotised reinforcement on its posterior surface pointed with a distinctive downward curve at apex and a broad, anterolateral, sclerotised acrophallus, on the anterior surface also curving downwards at the apex.

**Female**, as in male differing in the following traits: **Head**: bearing two pairs of proclinate orbital setae and two pairs of reclinate orbital setae, palpus dark ochraceous appearing brown to black, antennal pedicel dark orange almost brown, but distinctly lighter than postpedicel. **Abdomen**: overall appearing more globose than males and in its terminalia.

#### Diagnosis

*Santarosamyia erecta* (Coquillett, 1902) comb. nov. can be distinguished from its congeners by the apparent and thick vestiture of grey tomentum, which is gold in *S. woodorum* sp. nov. and the presence of four katepisternal setae, only three in *S. unipilum* comb. nov. It is distinguished from both of its congeners by its CO1 sequence.

#### Distribution

Widely distributed throughout North America, Canada (British Columbia, East, Ontario, Prairies) and USA (California, Florida, Great Plains, Northeast, Northern Rockies, Pacific Northwest, Southeast, Southwest, Texas) (O'Hara et al. 2020).

#### Ecology

*Nilea erecta* has been recorded parasitising larvae in the lepidopteran families: Tortricidae, Pyralidae, Noctuidae and Notodontidae (Arnaud 1978).

### Santarosamyia unipilum

(Aldrich & Webber, 1924) comb. nov.

1778248C-CF54-567D-B39D-A599B9C43A3E

unipilum Aldrich & Webber, 1924: 83 (Phorocera (Neopales)). Holotype male, (USNM) (Examined by: AJF and DMW). Type locality: USA, Oregon, Hood River.

#### Materials

- **Type status:** Other material. **Occurrence:** recordedBy: Borkent & Wood; individualID: Wood, D.M.; lifeStage: adult; preparations: pinned specimen; otherCatalogNumbers: 1751043; occurrenceID: 12E845CB-7831-5945-9833-7E41FE327CF4; **Taxon:** taxonID: Phorocera unipilum; scientificName: *Phorocera unipilum* Aldrich & Webber, 1924; order: Diptera; family: Tachinidae; genus: Phorocera; specificEpithet: unipilums; **Location:** country: Canada; countryCode: CA; stateProvince: Quebec; locality: Summit Rigaud Mountain; verbatimLocality: Quebec, Summit Rigaud Mountain; **Event:** eventDate: 1979-June-1; year: 1979; month: 06; day: 01; **Record Level:** collectionID: CNC
- **Type status:** Other material. **Occurrence:** recordedBy: Borkent & Wood; individualID: Wood, D.M.; lifeStage: adult; preparations: pinned specimen, dissected terminalia; occurrenceID: 33E77A4E-B2D0-5241-A0BE-1D8DF1422D9E; **Taxon:** taxonID: Phorocera unipilum; scientificName: *Phorocera unipilum* Aldrich & Webber, 1924; order: Diptera; family: Tachinidae; genus: Phorocera; specificEpithet: unipilum; **Location:** country: Canada; countryCode: CA; stateProvince: Nova Scotia; county: Kings County; verbatimLocality: KingsCo. N.S.; **Event:** eventDate: 1927-July-25; year: 1927; month: 07; day: 25; **Record Level:** collectionID: CNC
- **Type status:** Other material. **Occurrence:** recordedBy: Borkent & Wood; individualID: Wood, D.M.; sex: Female; lifeStage: adult; preparations: pinned specimen; otherCatalogNumbers: 1751043; occurrenceID: D78AB918-E340-5947-AC89-FAFC1E221BA8; **Taxon:** taxonID: Phorocera unipilum; scientificName: *Phorocera unipilum* Aldrich & Webber, 1924; order: Diptera; family: Tachinidae; genus: Phorocera; specificEpithet: unipilum; **Location:** country: Canada; countryCode: CA; stateProvince: Quebec; locality: Summit Rigaud Mountain; verbatimLocality: Quebec, Summit Rigaud Mountain; **Identification:** identifiedBy: D.M. Wood; **Event:** eventDate: 1979-June-1; year: 1979; month: 06; day: 01; **Record Level:** collectionID: CNC

#### Description

**Male** (Fig. 10), **Head**: head slightly wider than thorax when viewed dorsally; vertex 1/3 head width; gena 1/6 of head height, approximately 1/5 of eye height; with one row of frontal setae, these extending below base of pedicel and two pairs of reclinate orbital setae, nearly in line with frontal row; ocellar setae strong and proclinate, inserted directly adjacent to anterior ocellus; eye setulose; parafacial bare and narrow, slightly grey tomentose; gena grey, covered in short black setulae; fronto-orbital plate shining black, covered in black setulae surrounding frontal setae; lower margin of face level with vibrissa; facial ridge setose, along most of its length, almost reaching lowest frontal seta; pedicel black; postpedicel black, 4x as long as pedicel; arista bare, distinctly-thickened on basal half. Palpus dark brown, almost black, densely setulose, digitiform, not distinctly clubbed.

**Thorax**: scutum black ground colour, apparently glabrous devoid of tomentum, with some sparse grey microtomentum visible under oblique angled light; four dorsal vittae, almost indistinct, becoming slightly more prominent under certain angles of light; postpronotum bearing four setae, middle basal seta in line with outer and inner basal setae; anterior margin of anipemeron with only 2–4 long setae. Chaetotaxy: acrostichal setae 3:3; dorsocentral setae 3:4; intra-alar setae 3:3; supra-alar setae 2:3; original description cites three katepisternal setae; however, examination of the holotype and specimens from Quebec and Nova Scotia suggest four katepisternal setae; scutellum black with dark maroon along basal edges, with one pair of discal setae and three pairs of long flat marginal setae.

**Abdomen**: ground colour black; abdominal tomentum apparently absent, grey microtomentum visible under certain angles of light becoming more dense laterally; median discal setae present T3 and T4.

**Male terminalia** (Fig. 11): sternite 5 with a deeply excavated median cleft along posterior edge, approximately 2s as wide as long, V-shaped, inner margins covered in dense tomentum; posterior lobes pointed and vaguely triangular apically, two strong setae surrounded by many shorter, weaker setulae; unsclerotised "window" on anterior plate of sternite 5 almost entirely translucent, distinct from posterior lobes. Epandrium setulose, cercus triangular, slightly longer than surstyli; cercus apically pointed, completely separate along most of their length. Cercus in lateral view, with a slight downward curve at apex, densely setulose along basal 2/3. Surstylus in lateral view, wide and robust, round medially, rounded and blunt at apex, not tapering to a point, giving the structure a wide digitate appearance; surstylus not fused with epandrium; when viewed dorsally, surstyli wide, slightly divergent, bearing a slight outward bend at apices. Pregonite broad, well-developed, apically rounded, blunt, with 6–7 setae along margin. Postgonite, narrowed, up to 1/2 as wide as pregonite, curved at apex. Basiphallus with a well-developed narrow and curved epiphallus, distiphallus broad with a thick median longitudinal sclerotised reinforcement on its posterior surface pointed with a distinctive downward curve at apex and a broad, anterolateral, sclerotised acrophallus, on the anterior surface curving upwards at the apex.

**Female**, as in male differing in the following traits: **Head**: bearing two pairs of proclinate orbital setae and two pairs of reclinate orbital setae, palpus dark ochraceous appearing brown to black, pedicel dark brown. **Thorax**: in some cases, females appear to possess three katepisternal setae, but this trait is variable. **Abdomen**: slightly more globose than males and in its terminalia.

#### Diagnosis

*Santarosamyia unipilum* (Aldrich and Webber 1924) comb. nov. can be distinguished from its congeners *S. erecta* comb. nov. and *S. woodorum* sp. nov. by the sparse vestiture of grey tomentum, giving the overall appearance of glabrous black on the thorax and abdomen and by its CO1 sequence.

#### Distribution

North America, Canada (Ontario, Quebec, Maritimes recorded by D.M. Wood), USA (Pacific Northwest) (O'Hara et al. 2020).

#### Ecology

No host information is available for *Santarosamyia unipilum*.

## Identification Keys

### Key to the new genus *Santarosamyia*

**Table:** 

1	Thorax and abdomen with thick grey or slightly golden tomentum	2
–	Thorax and abdomen appearing glabrous black, if tomentum is visible, then only as microtomentum under certain angles of light	*Santarosamyia unipilum* (Reinhard, 1922) **comb. nov.**
2	Thorax and abdomen yellow-gold tomentose, thorax with four katepisternal setae	*Santarosamyia woodorum* **sp. nov.**
–	Thorax and abdomen grey tomentose, thorax with three katepisternal setae	*Santarosamyia erecta* (Coquillett, 1902) **comb. nov.**

## Discussion

In the most recent treatise on the subject of the tribe Eryciini, Seyyedi-Sahebari et al. (2021) indicated the following combination of characters as generally useful in recognition of genera within the tribe: prosternum setose, katepimeron bare, first postsutural supra-alar setae at least as long and stout as notopleural and first postsutural intra-alar setae, mid-dorsal depression on syntergite 1+2 reaching hind margin of the segment, postpronotum with fewer than five setae, katepimeron bare or at most with few sparse setae, hind tibia irregularly setose, vibrissa arising level with facial margin, inner margin of lower calypter curving away from scutellum, gena wider than profrons, antenna inserted around or above the middle of eye and ocellar setae never reclinate, to which we can also add eyes densely haired, but even these characters have exceptions within the tribe. Despite this research, there remains no definitive diagnosis of the tribe Eryciini (and “questionable monophyly” (Stireman 2002, Stireman et al. 2019) due to its wide morphological variation, lacking any very positive synapomorphies for tribal definition. The one notable exception is a trend towards ovo-larvipary directly on the host integument, separating it from its sister tribe, the Goniini, which lays microtype eggs on the host plant (Stireman 2002, Stireman et al. 2019).

The establishment of *Santarosamyia* as a valid genus is supported by multiple lines of evidence that clearly separate it from the related genera *Nilea* Robineau-Desvoidy and *Lespesia* Coquillett. In the key by Wood (1987), the new genus *Santarosamyia* runs to couplets 43 and 46 alongside *Nilea*, from which it is distinguished by the distinct morphology of the distiphallus. In the more recent key by Wood and Zumbado (2010), it runs to couplet 57 with *Lespesia*, from which it is distinguished by the presence of discal setae on tergites 3 and 4 and by the distinct morphology of the distiphallus. As illustrated in Fig. 12, these differences include the curvature of the median ventral process of the distiphallus, the form of the postgonite and the shape of the cercus. These morphological differences of the male postabdomen are corroborated by the molecular data, showing clear distinctions between *Santarosamyia* and *Nilea innoxia* R.-D.

A comparison of DNA barcodes provides a compelling example of this separation. An analysis using publicly available sequence data reveals that, on average, *Santarosamyia* differs from *Nilea* by 46 base pairs in the standard barcoding region. A significant number of these nucleotide changes are non-synonymous substitutions, resulting in an average of seven amino acid differences between the two groups. These amino acid differences are indicative of much deeper and older differentiation than would be expected between species of the same genus. While COI divergence is not always a definitive phylogenetic signal — as good species can either share DNA barcodes or be characterised by much greater divergences — the magnitude of divergence observed here is highly informative. Such a substantial molecular gap, especially when correlated with the consistent morphological discontinuities noted, provides robust evidence that these lineages are not congeneric.

Although additional research is needed to determine its closest relatives within the Eryciini, the available morphological and DNA barcode data confirm that *Santarosamyia* is distinctly divergent from similar taxa like *Nilea* and *Lespesia*. This level of divergence provides a robust justification for the erection of a new genus, consistent with the criteria applied in similar taxonomic studies (as in Sterling et al. (2023)).

## Supplementary Material

XML Treatment for Santarosamyia

XML Treatment for Santarosamyia woodorum

XML Treatment for Santarosamyia erecta

XML Treatment for Santarosamyia unipilum

24D85790-5F61-590A-ACA7-C3D3B0665D3C 10.3897/BDJ.13.e161853.suppl1 Supplementary material 1 Figure 3 Sample Information Data type table Brief description Supplemental table X containing metadata associated with samples in Figure 3. File: oo_1402648.xlsx https://binary.pensoft.net/file/1402648 M. Alex Smith

C50AFC32-5664-52FC-8DAD-39A0FF9F02E7 10.3897/BDJ.13.e161853.suppl2 Supplementary material 2 Figure 3 Sample information 2 Data type table Brief description Supplemental Table Y pairwise intergeneric percentage of base substitutions per site derived by averaging over all sequence pairs between genera. File: oo_1402649.xlsx https://binary.pensoft.net/file/1402649 M. Alex Smith

3C181B4D-E8AB-557F-81F5-F38CD1E06095 10.3897/BDJ.13.e161853.suppl3 Supplementary material 3 Figure 4 Sample Information Data type table Brief description Supplemental table Z containing metadata associated the samples in Figure 4. File: oo_1402650.xlsx https://binary.pensoft.net/file/1402650 M. Alex Smith

## Figures and Tables

**Figure 1a.**
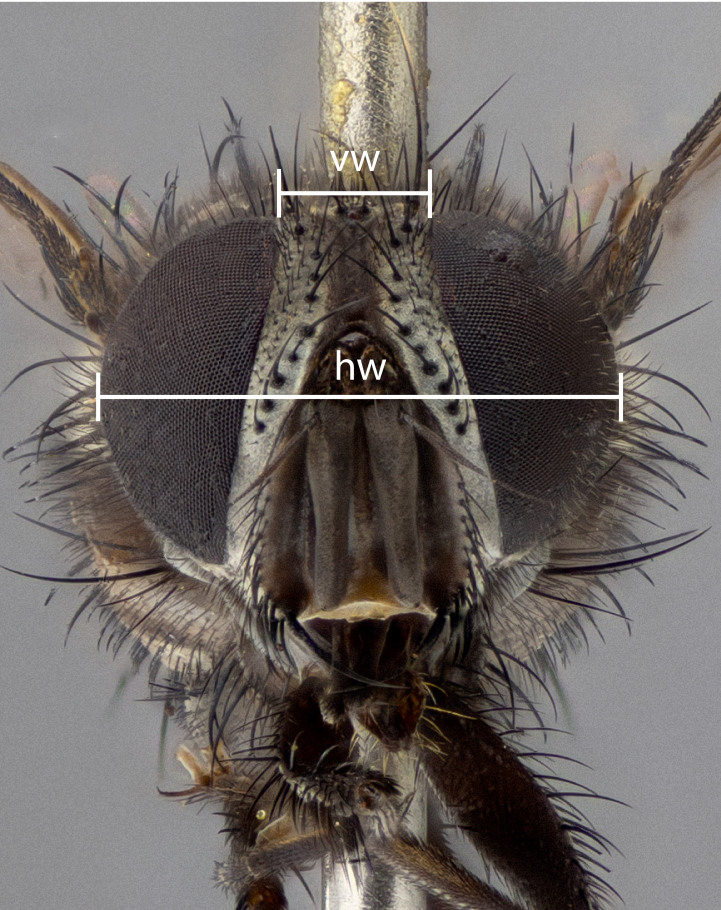
sample of measured areas from front of head as shown on *S. woodorum* **sp. nov.**; vw = vertex width; hw, head width;

**Figure 1b.**
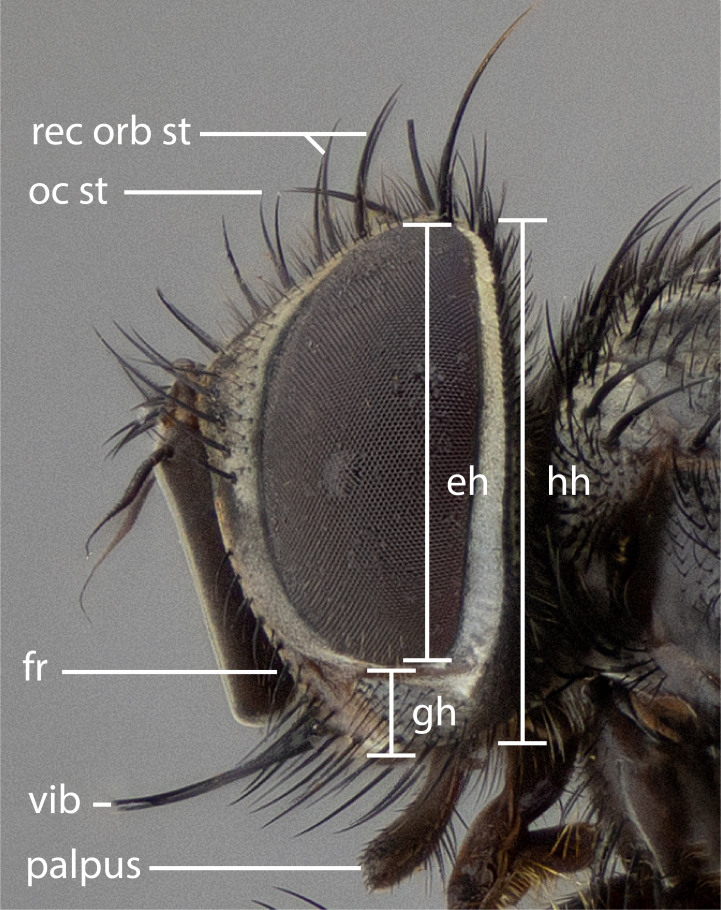
sample of measured areas from profile of head as shown on *S. woodorum* **sp. nov.**; eh = eye height, fr = facial ridge, gh = genal height, hh = head height, oc st = ocellar seta, palpus = palpus, rec orb st = reclinate orbital seta, vib = vibrissa.

**Figure 2a.**
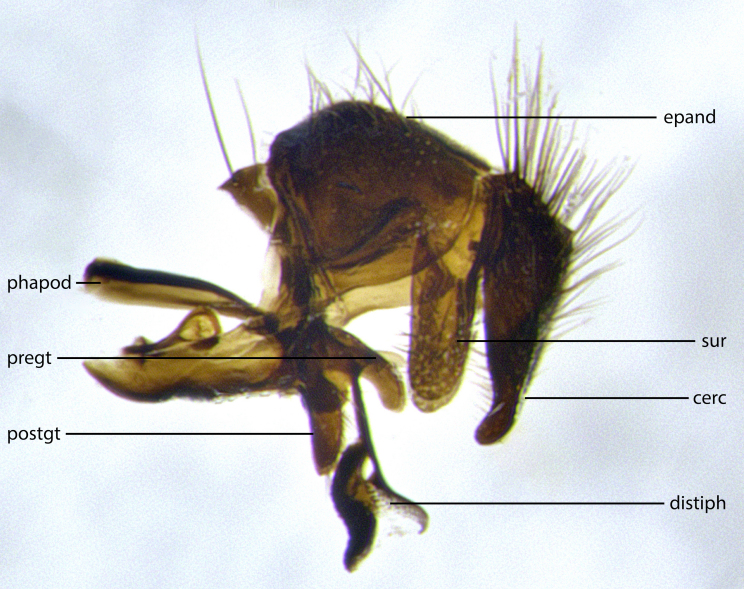
lateral view of terminalia of *Santarosamyia woodorum* **sp. nov.**; abbreviations: cerc = cercus; distph = distiphallus; epand = epandrium; hypd = hypandrium; phapod = phalloapodeme; pgt = postgonite; pregt = pregonite; sur = surstylus;

**Figure 2b.**
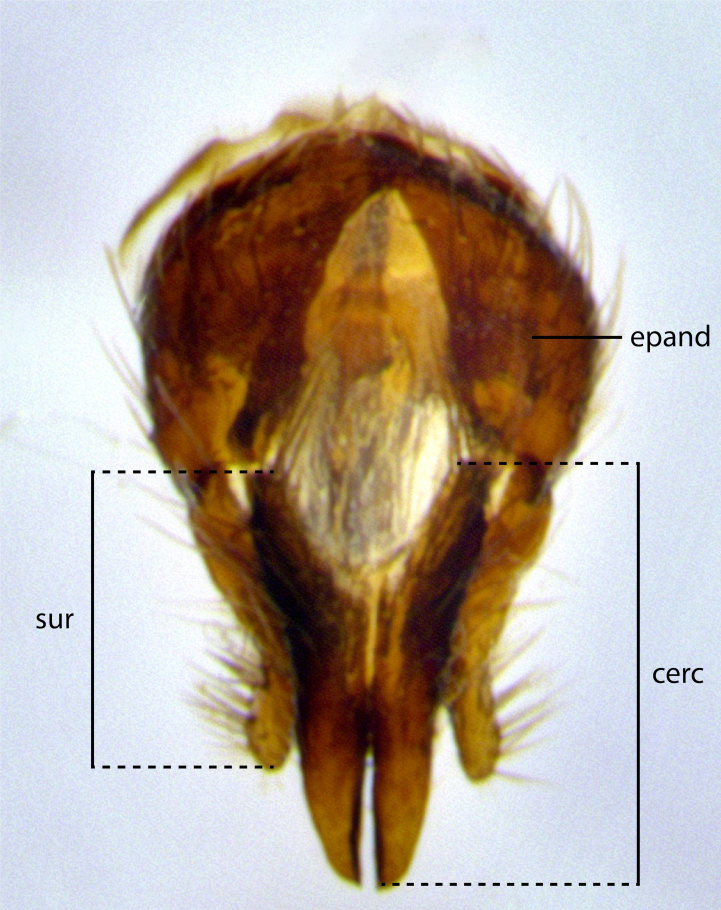
dorsal view of terminalia of *S. unipilum* **comb. nov.**; abbreviations for sections measured: cerc = cercus; epand = epandrium; sur = surstylus;

**Figure 2c.**
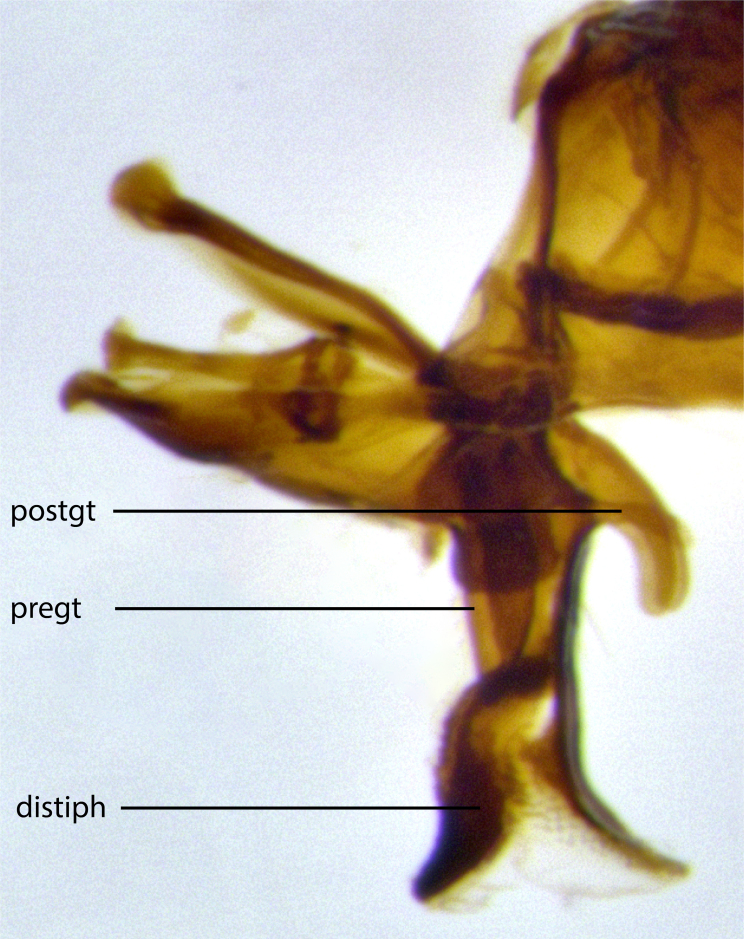
detailed view of adeagus *S. unipilum* **comb. nov.**; abbreviations: distph = distiphallus; pgt = postgonite; pregt = pregonite;

**Figure 2d.**
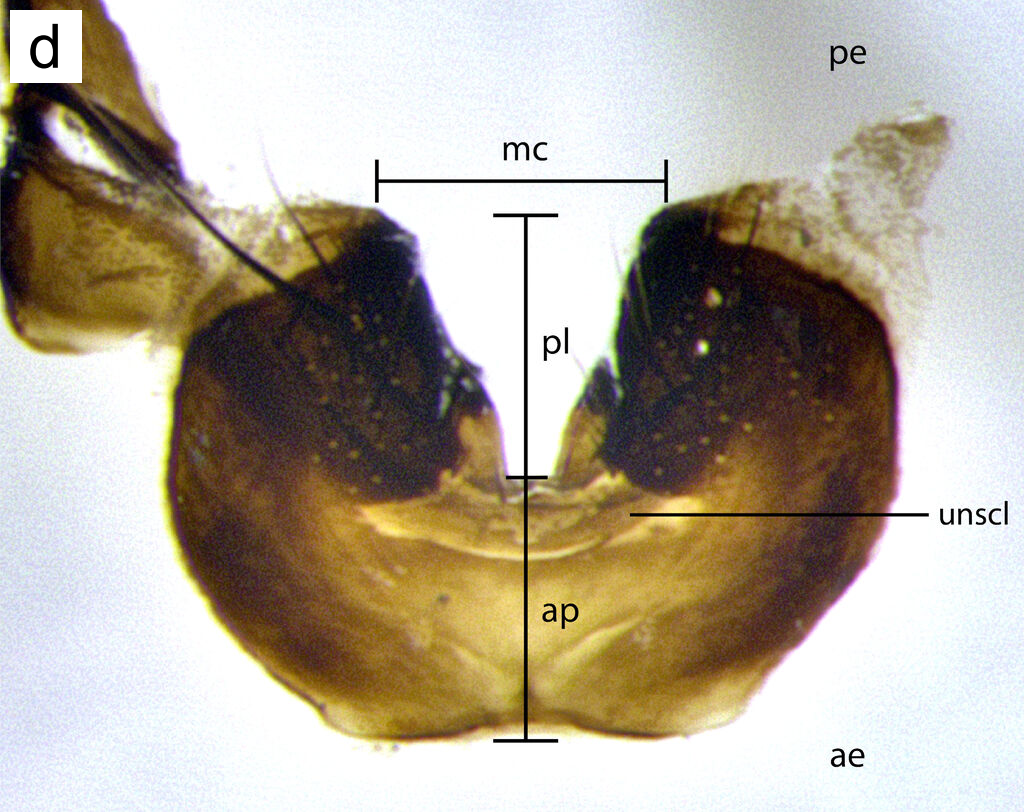
ventral view of sternite 5 *S. woodorum* **sp. nov.**; abbreviations: ae = anterior edge; ap = anterior plate; mc = median cleft; pe = posterior edge; pl = posterior lobes.

**Figure 5a.**
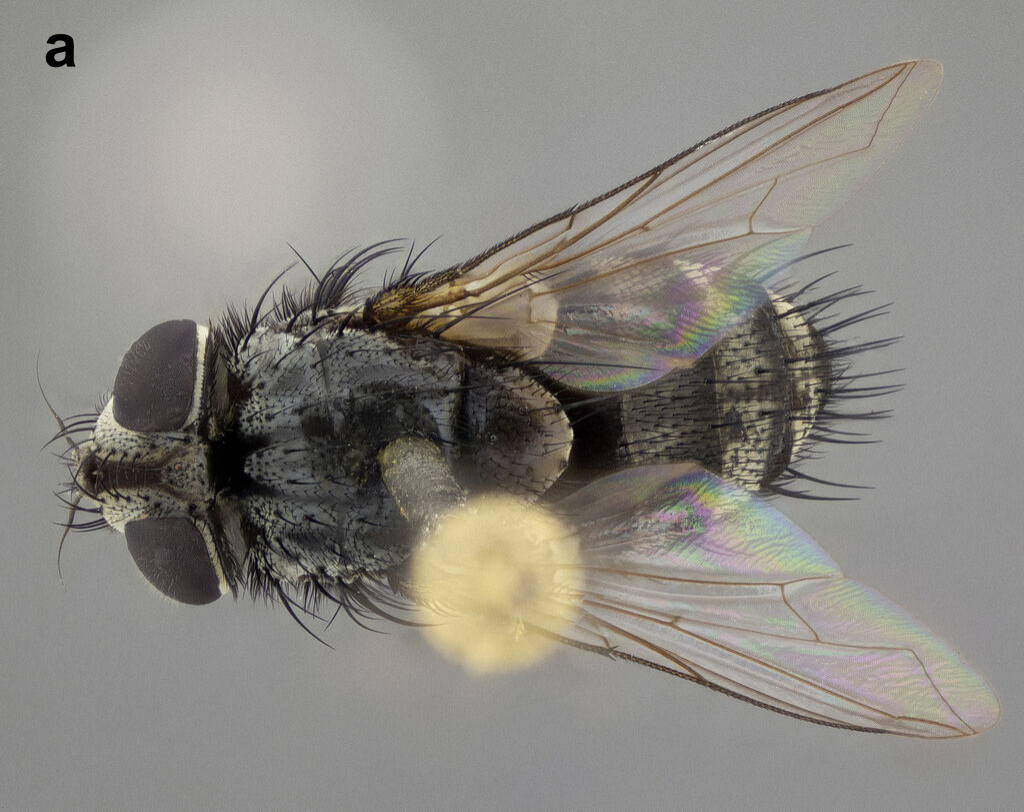
dorsal view;

**Figure 5b.**
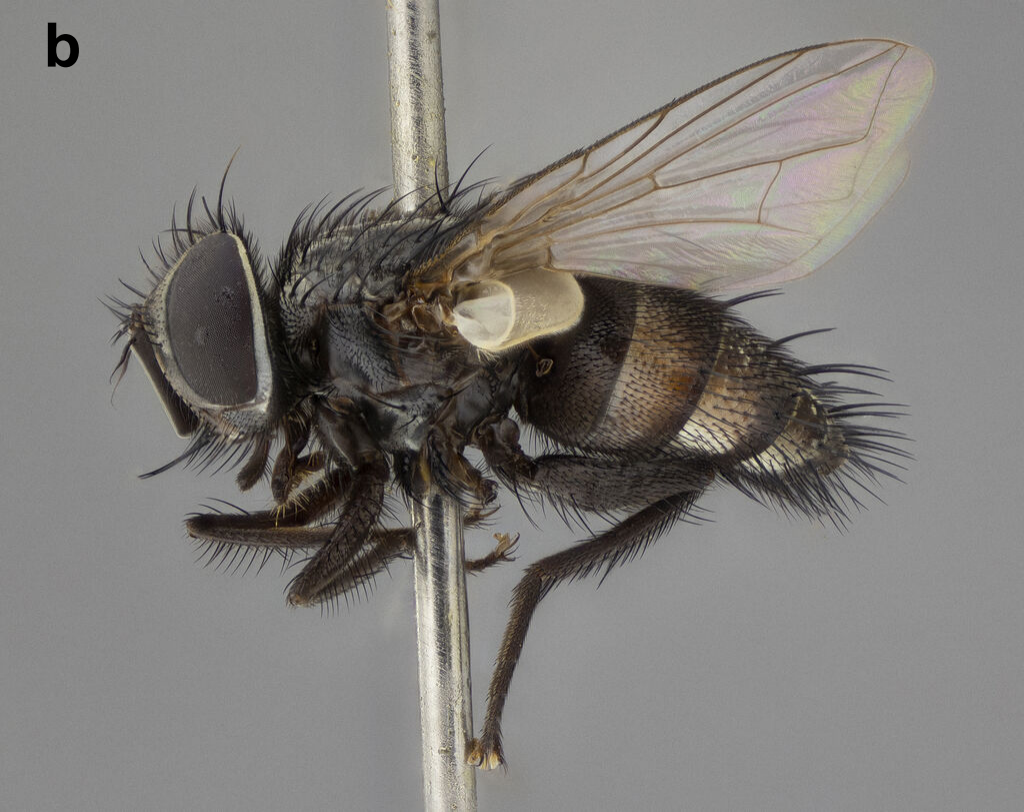
lateral view;

**Figure 5c.**
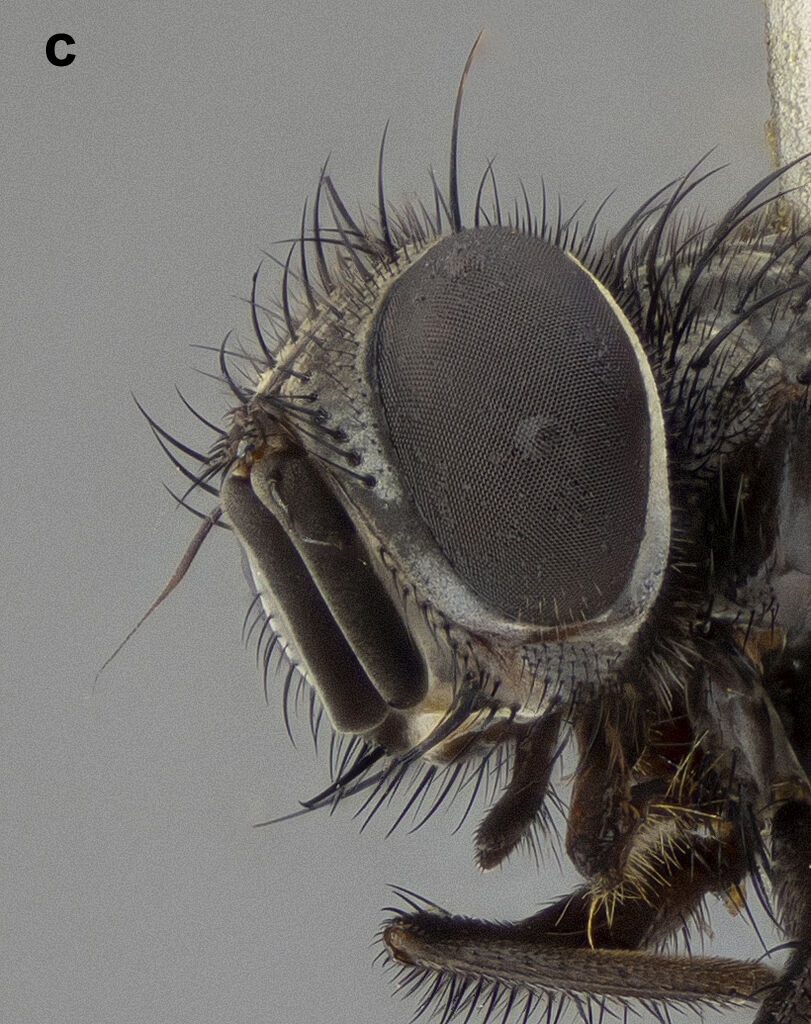
three-quarters view;

**Figure 5d.**
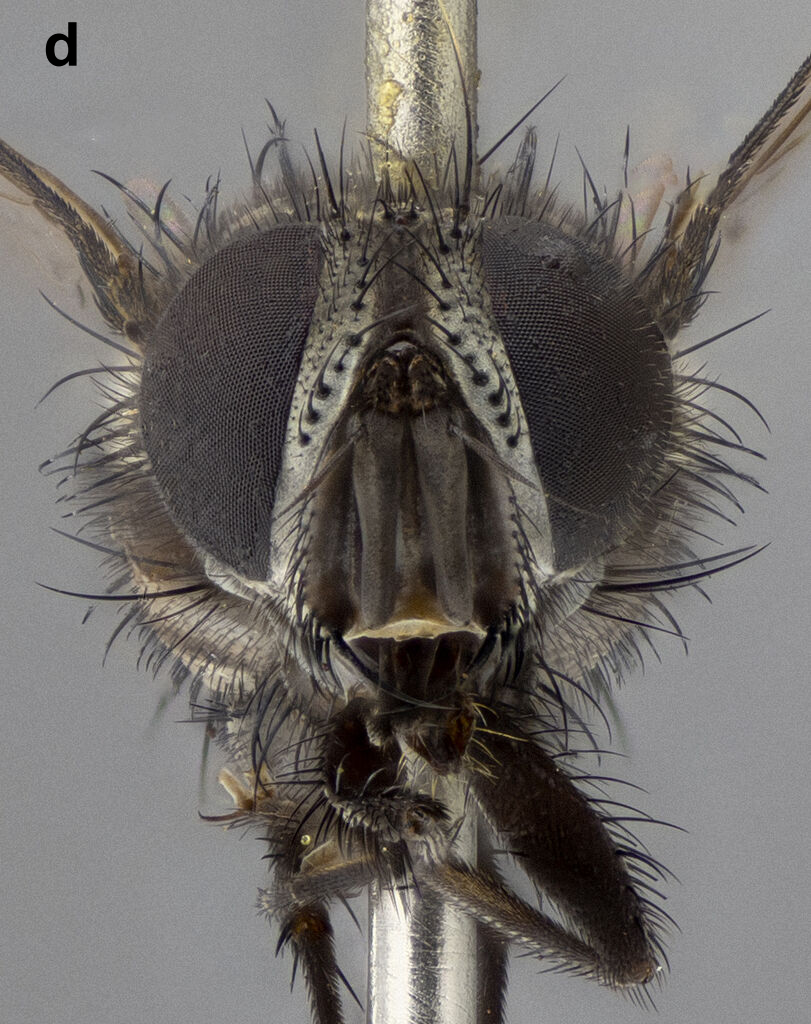
frontal view.

**Figure 6a.**
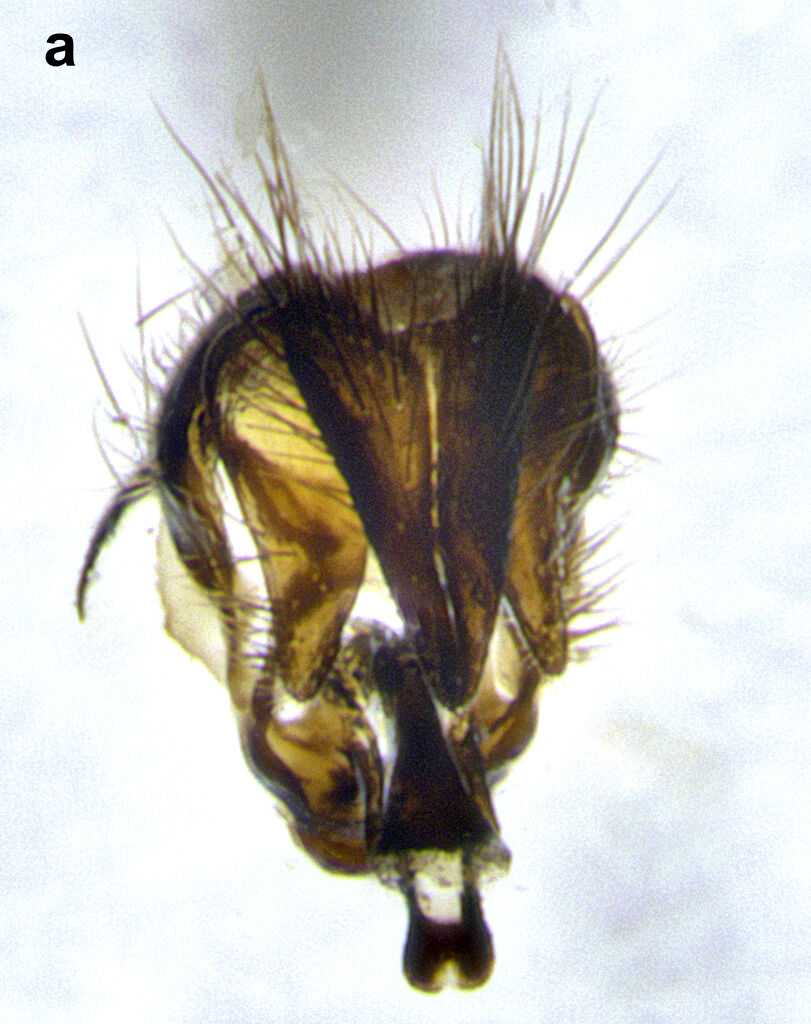
caudal view;

**Figure 6b.**
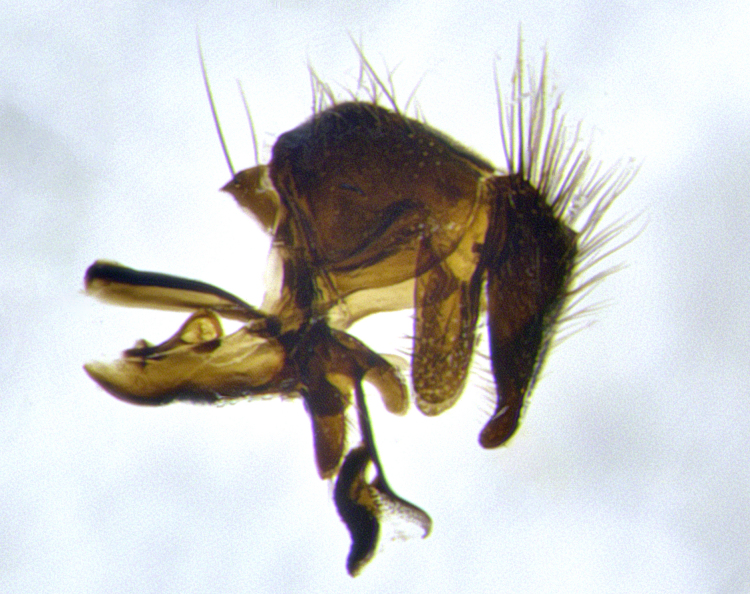
lateral view;

**Figure 6c.**
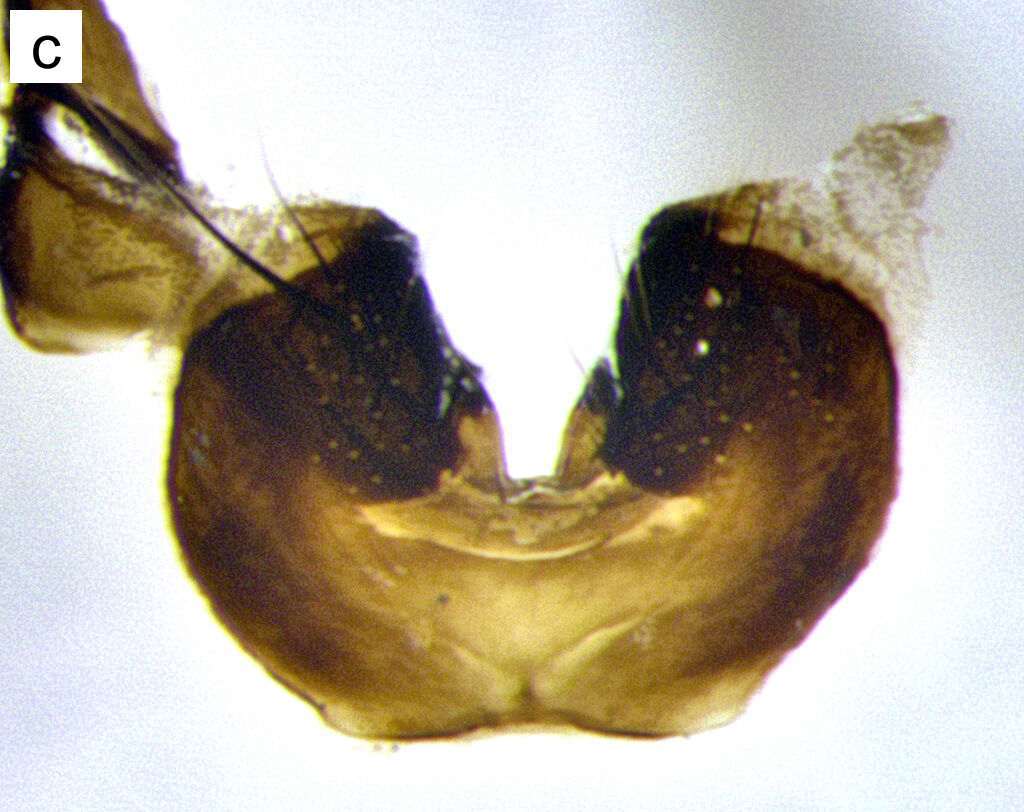
sternite 5;

**Figure 6d.**
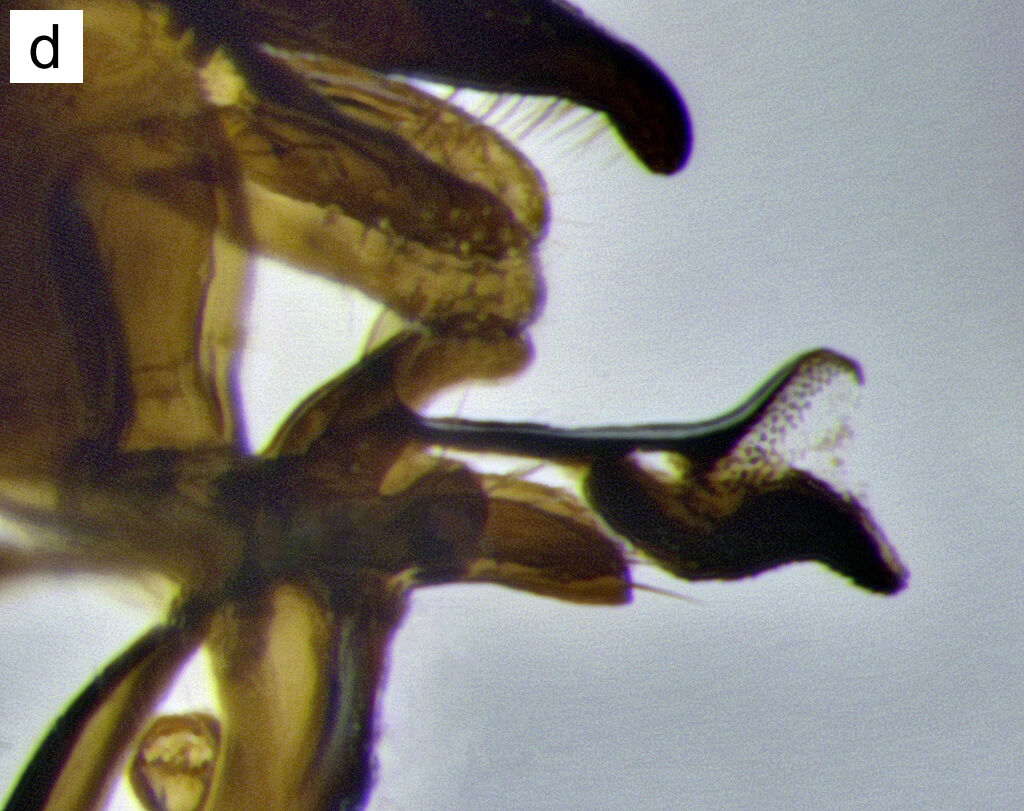
detailed lateral view of distiphallus.

**Figure 7a.**
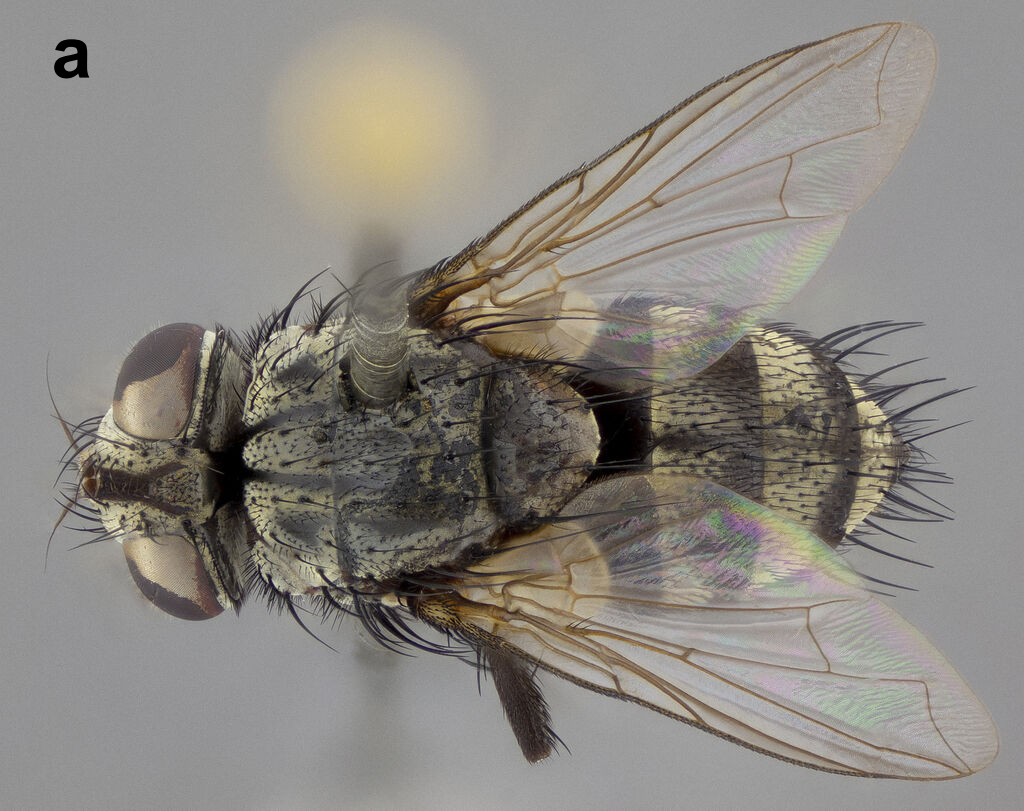
dorsal view;

**Figure 7b.**
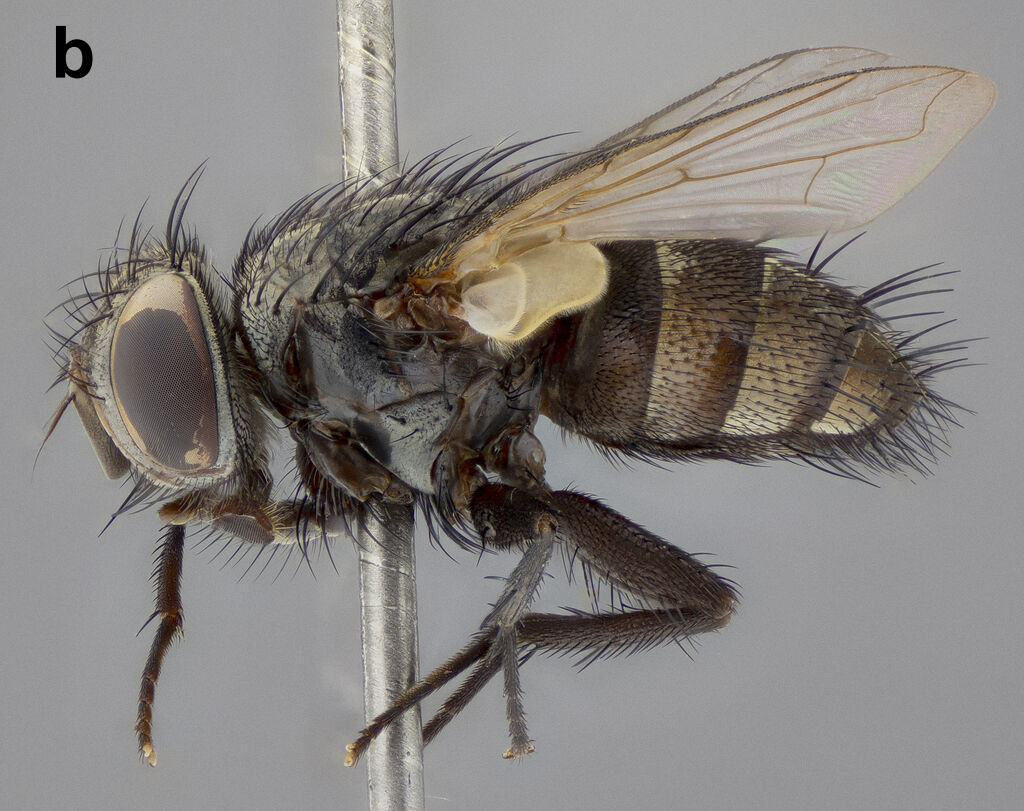
lateral view;

**Figure 7c.**
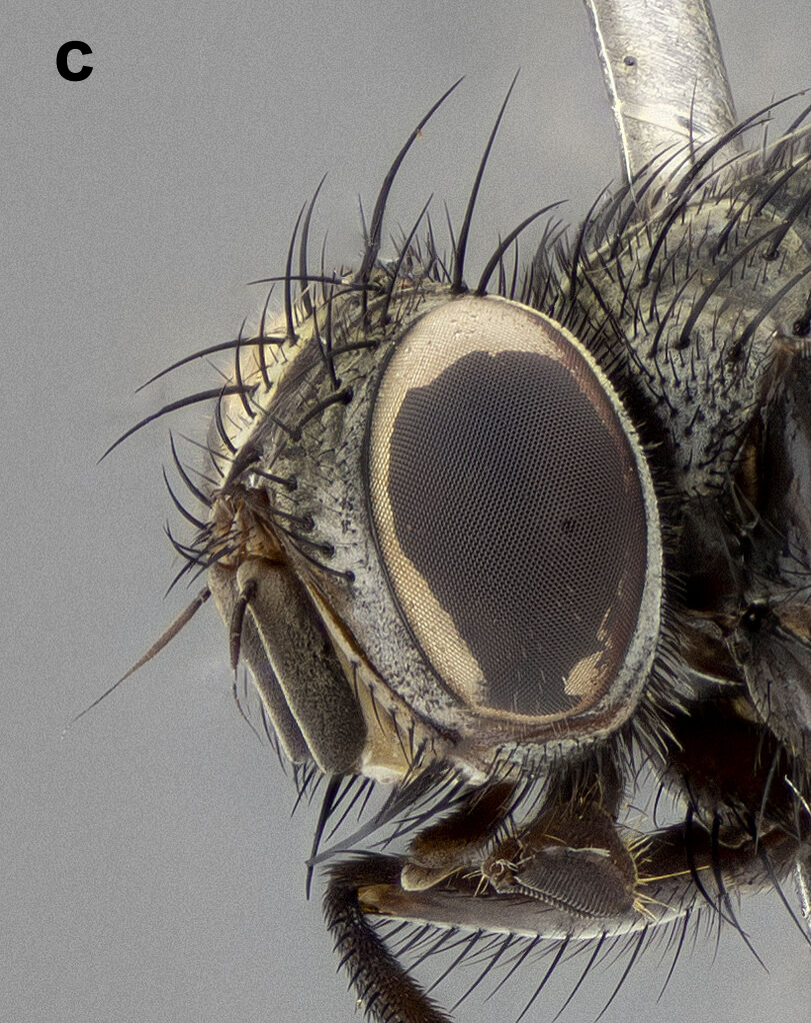
three-quarters view;

**Figure 7d.**
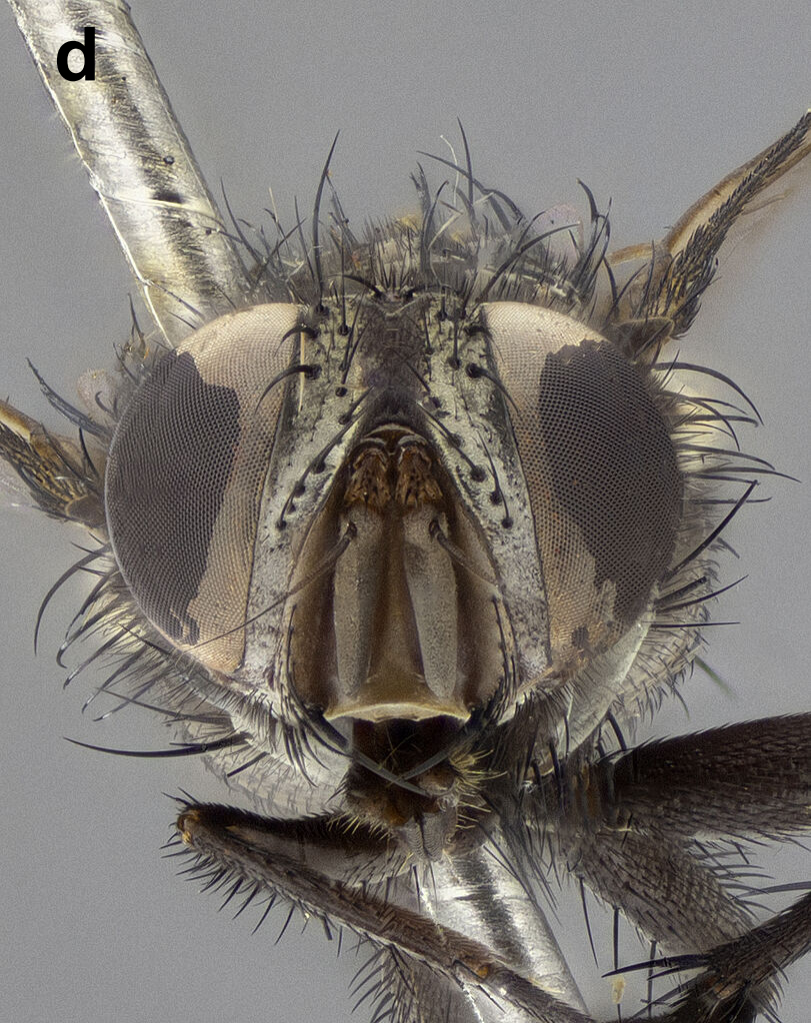
frontal view.

**Figure 8a.**
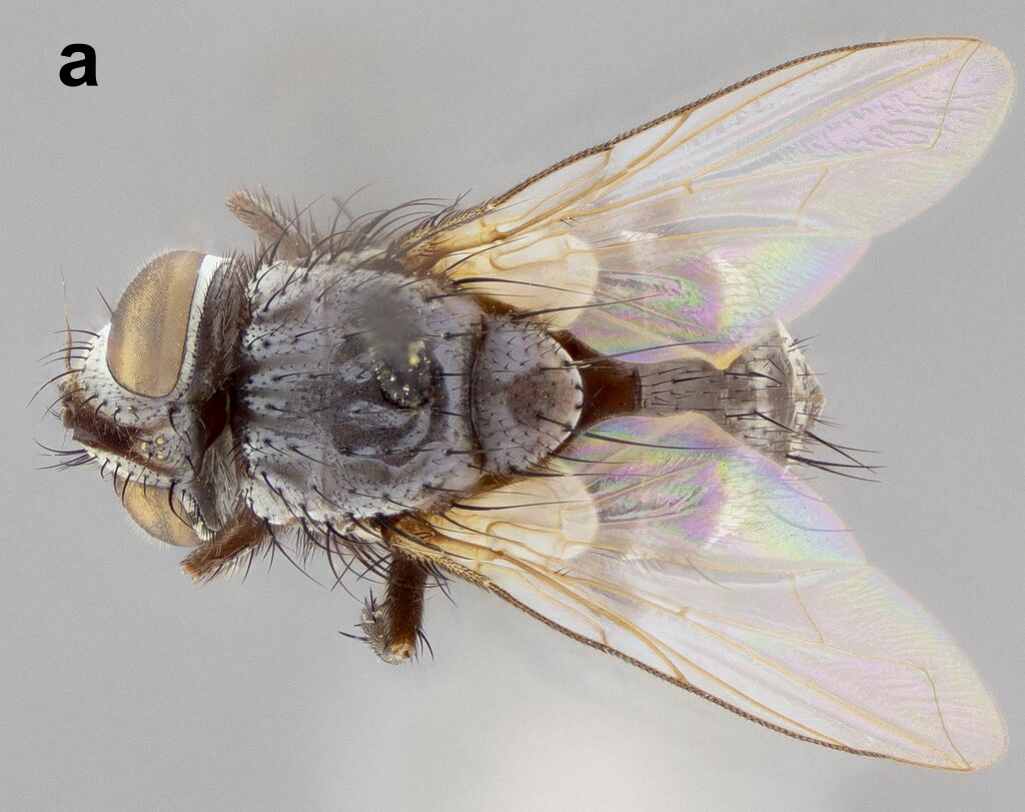
dorsal view;

**Figure 8b.**
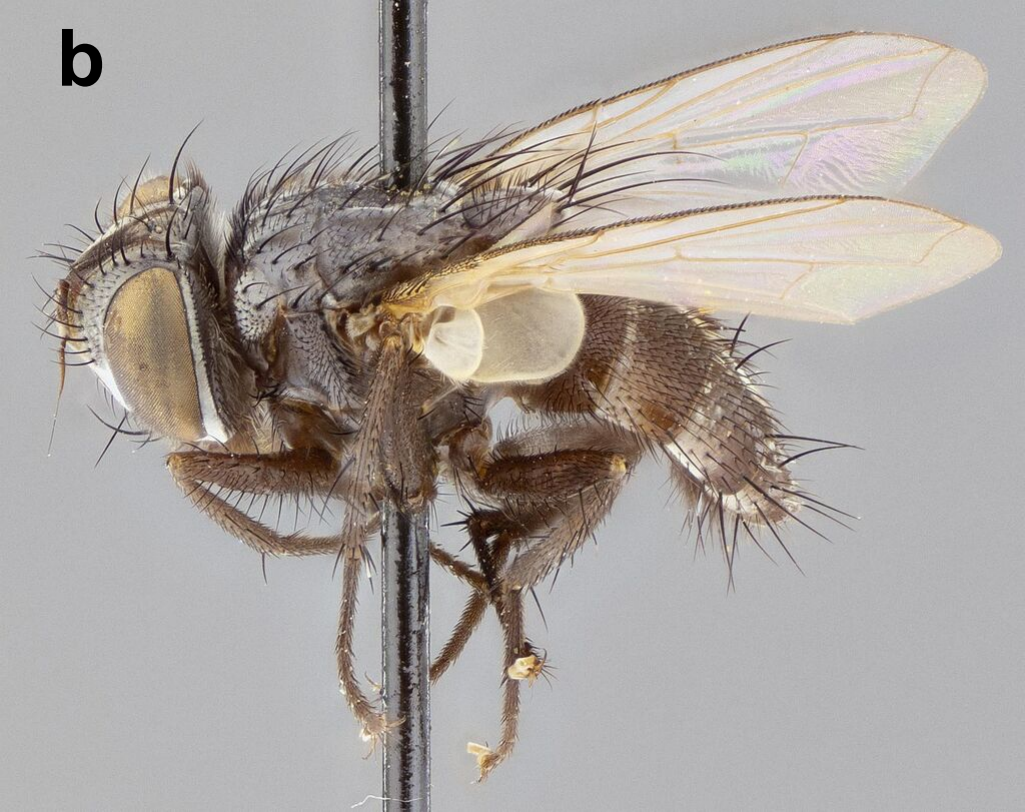
lateral view;

**Figure 8c.**
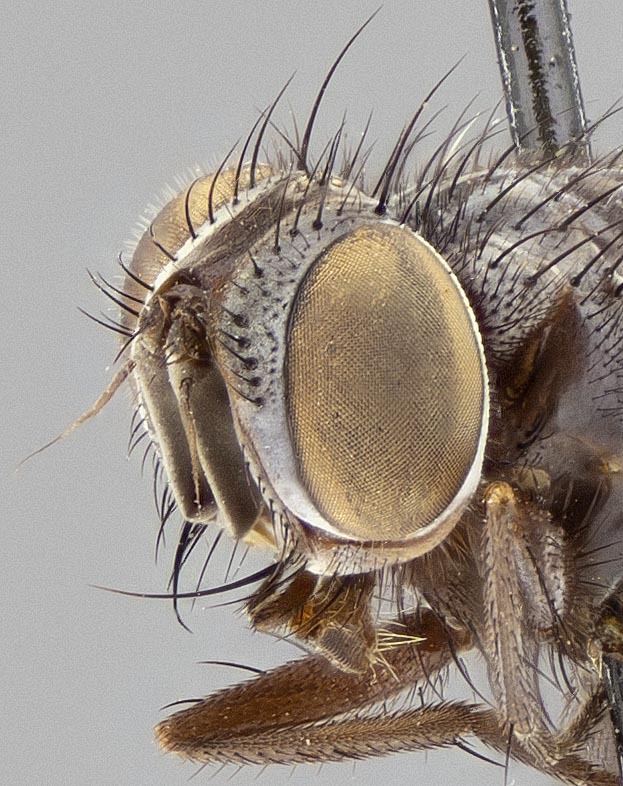
three-quarters view;

**Figure 8d.**
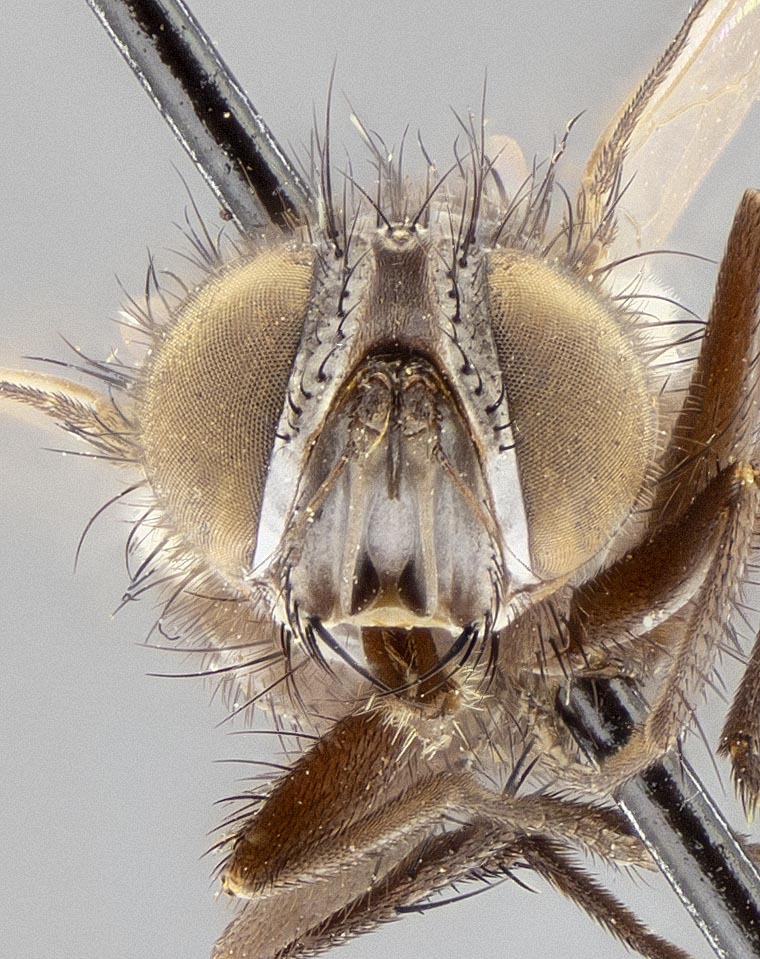
frontal view.

**Figure 9a.**
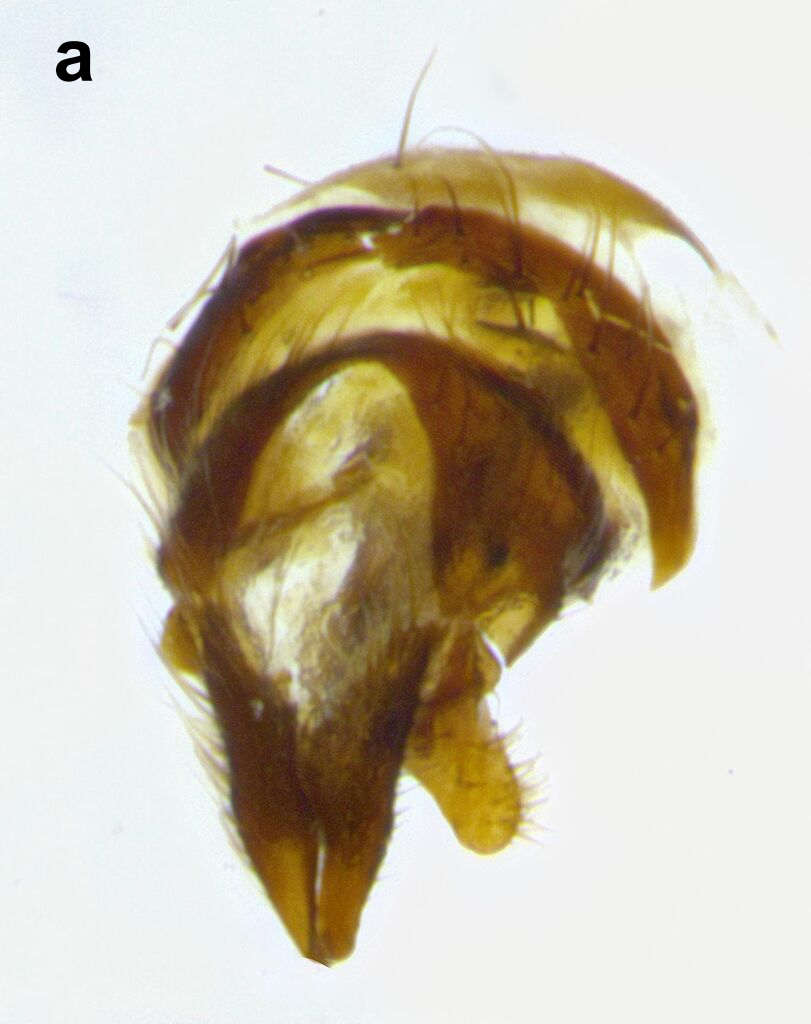
caudal view;

**Figure 9b.**
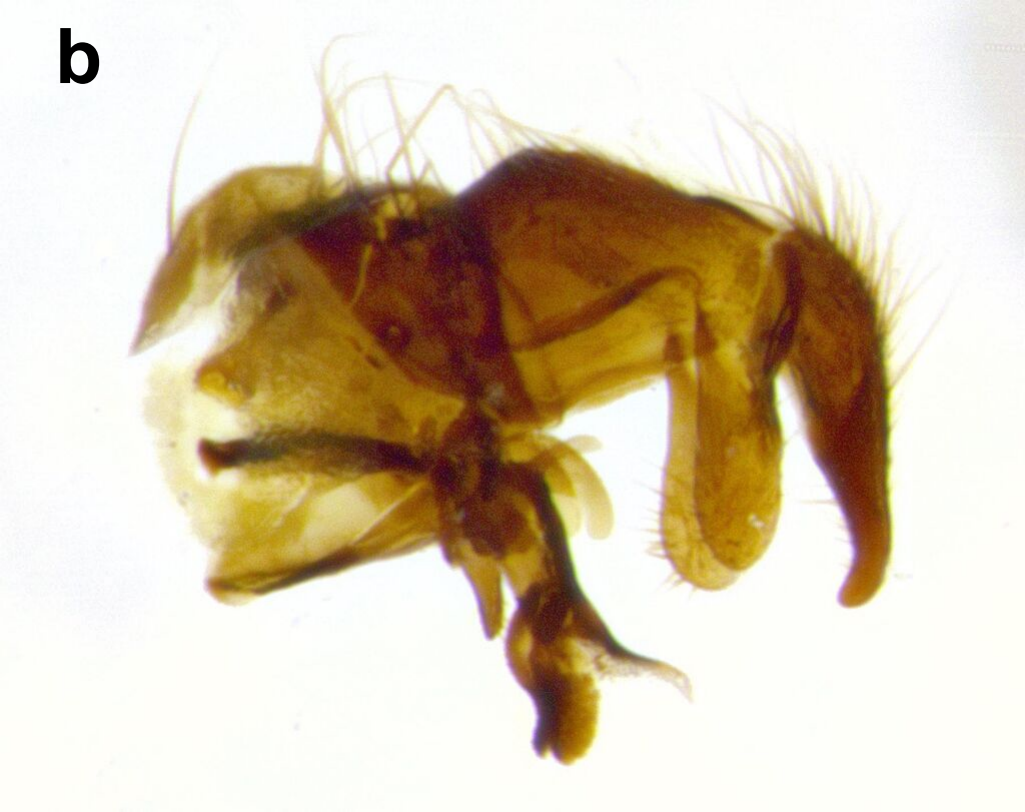
lateral view;

**Figure 9c.**
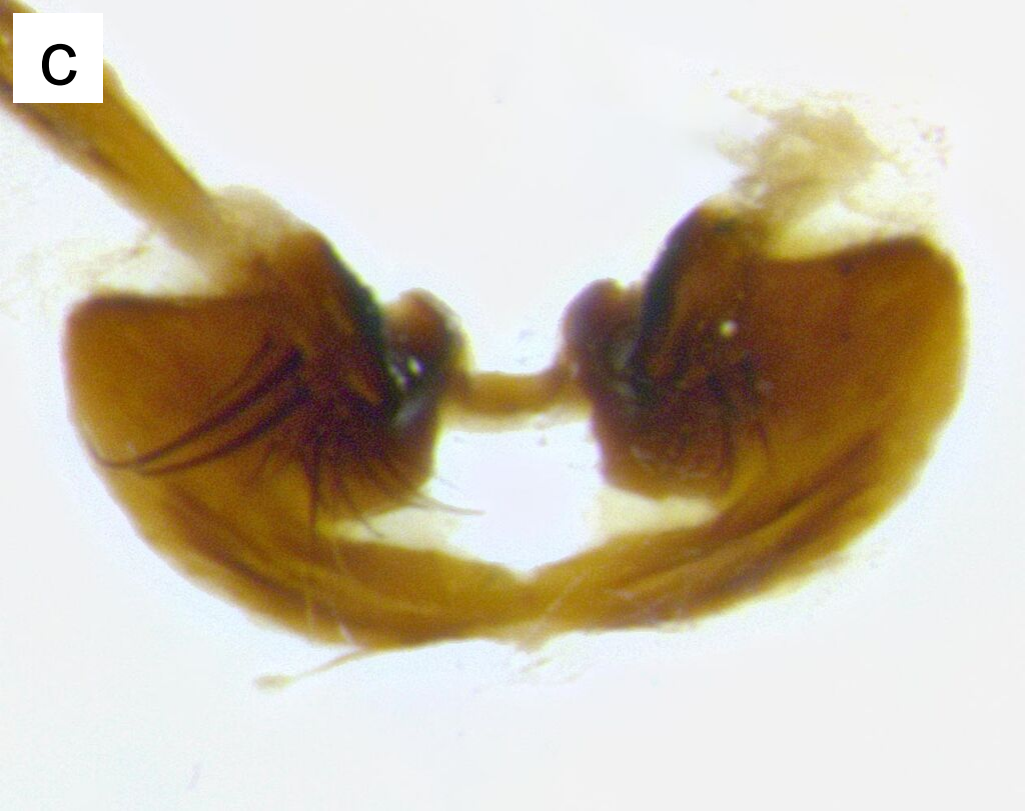
sternite 5;

**Figure 9d.**
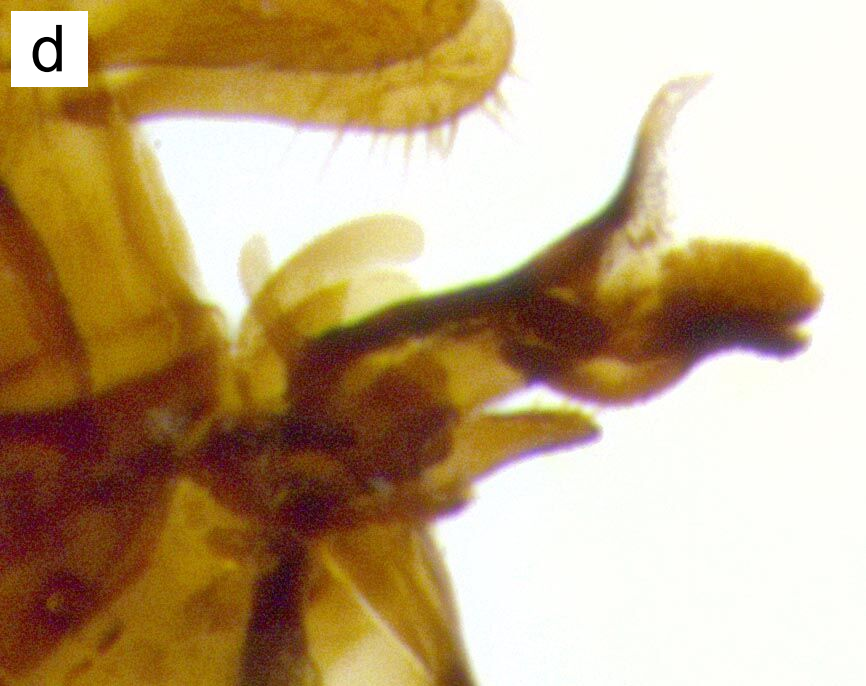
detailed lateral view of distiphallus.

**Figure 10a.**
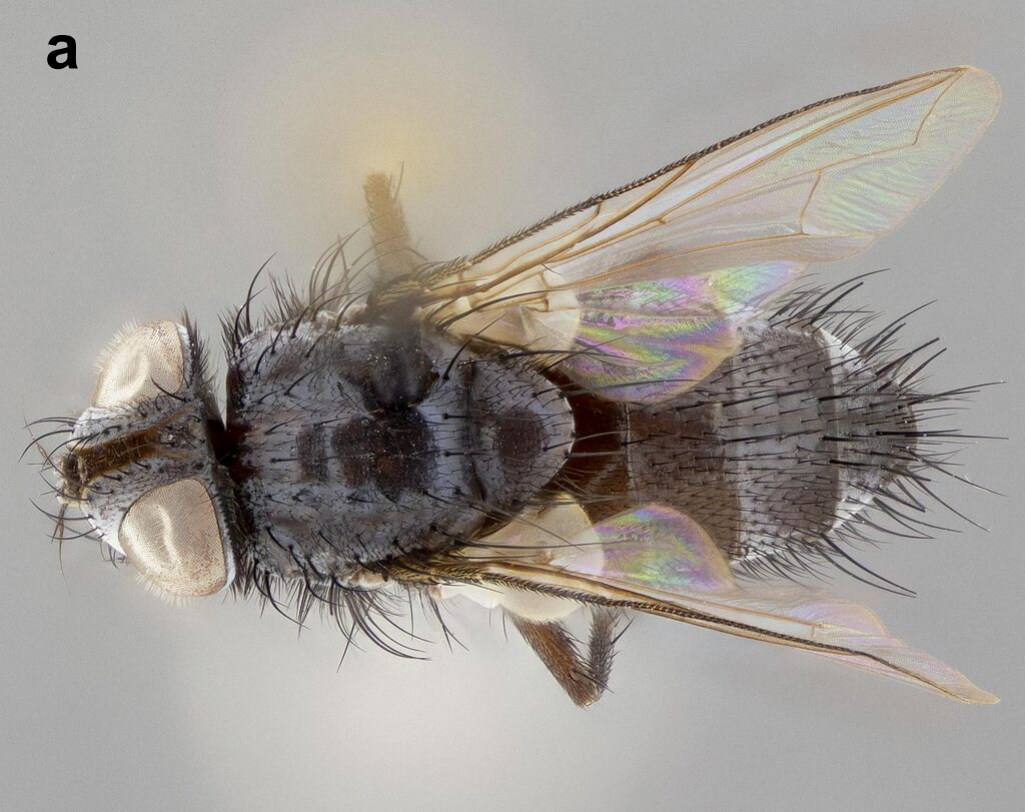
dorsal view;

**Figure 10b.**
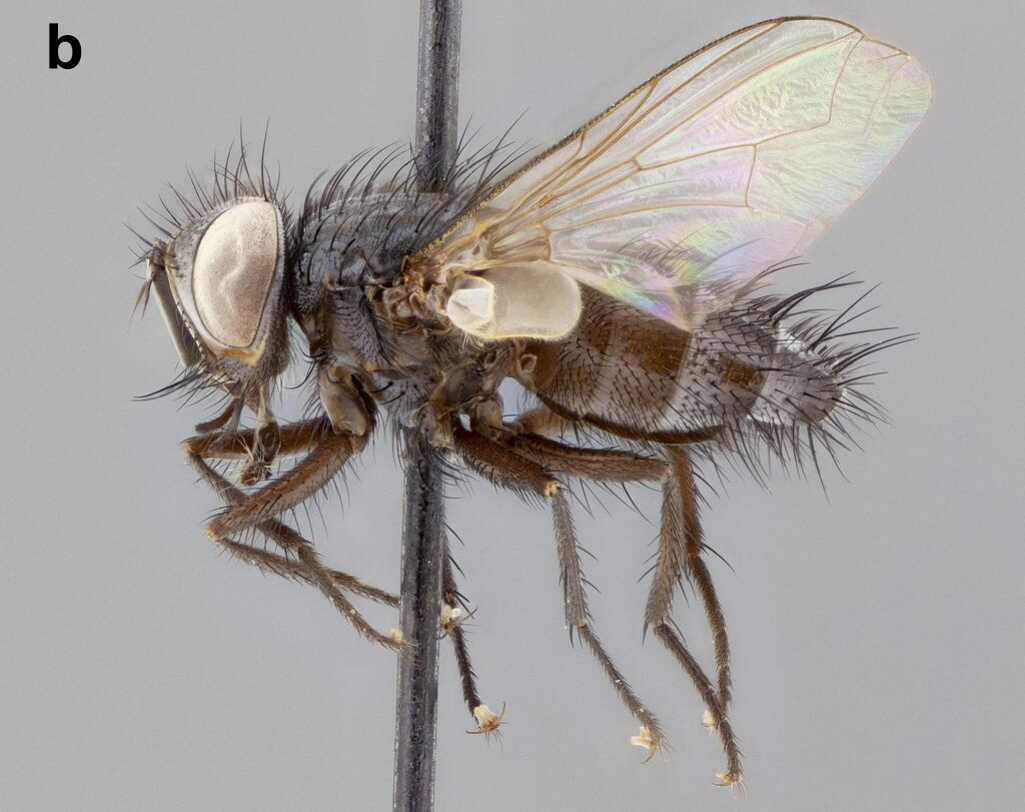
lateral view;

**Figure 10c.**
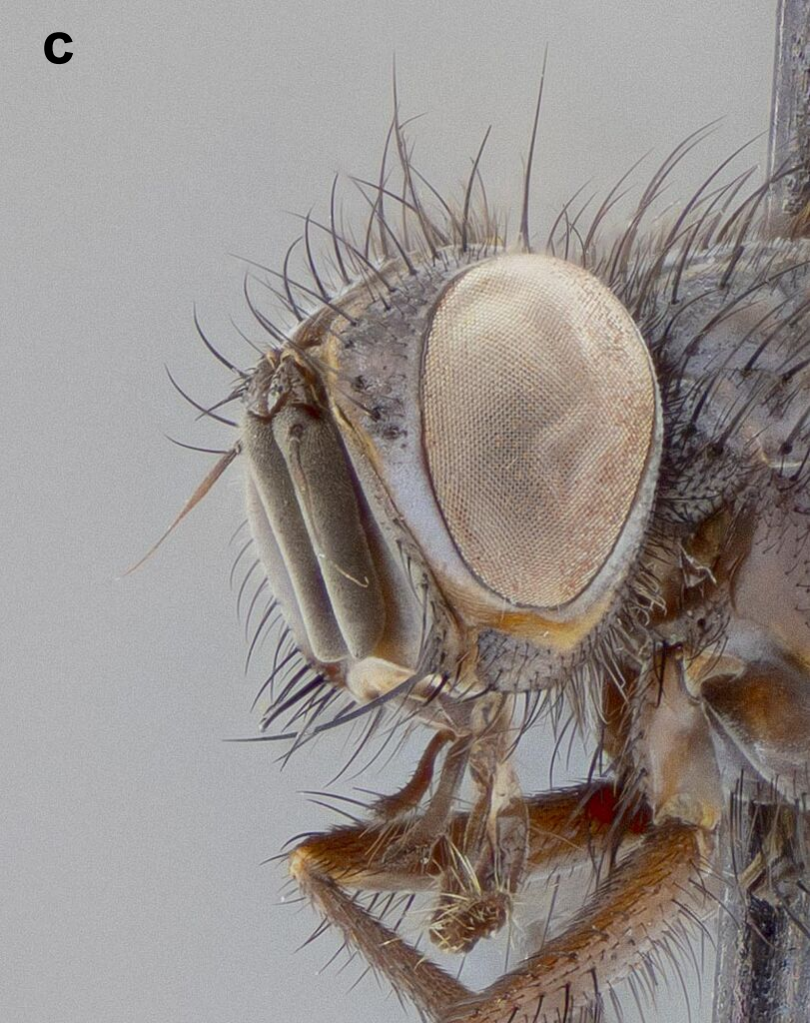
three-quarters view;

**Figure 10d.**
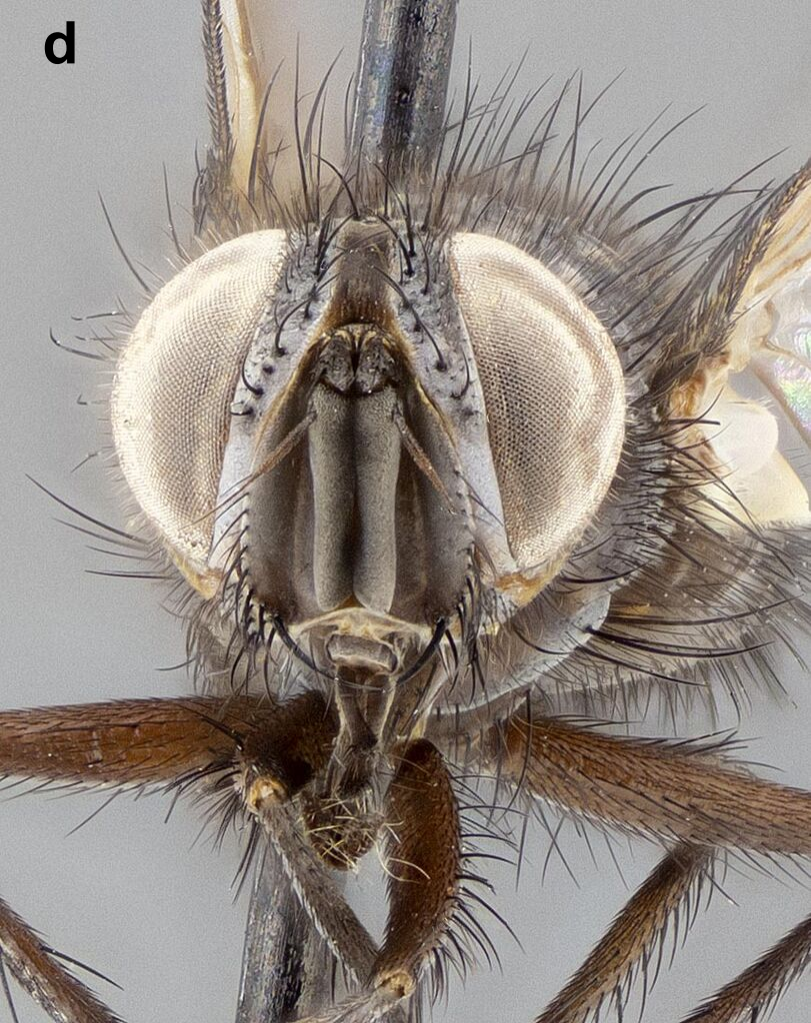
frontal view.

**Figure 11a.**
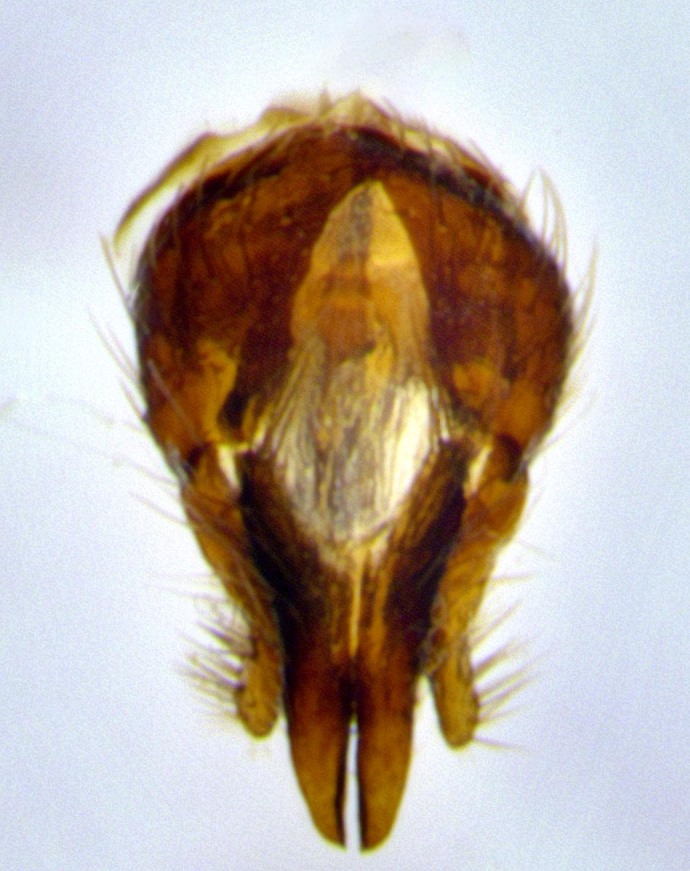
caudal view;

**Figure 11b.**
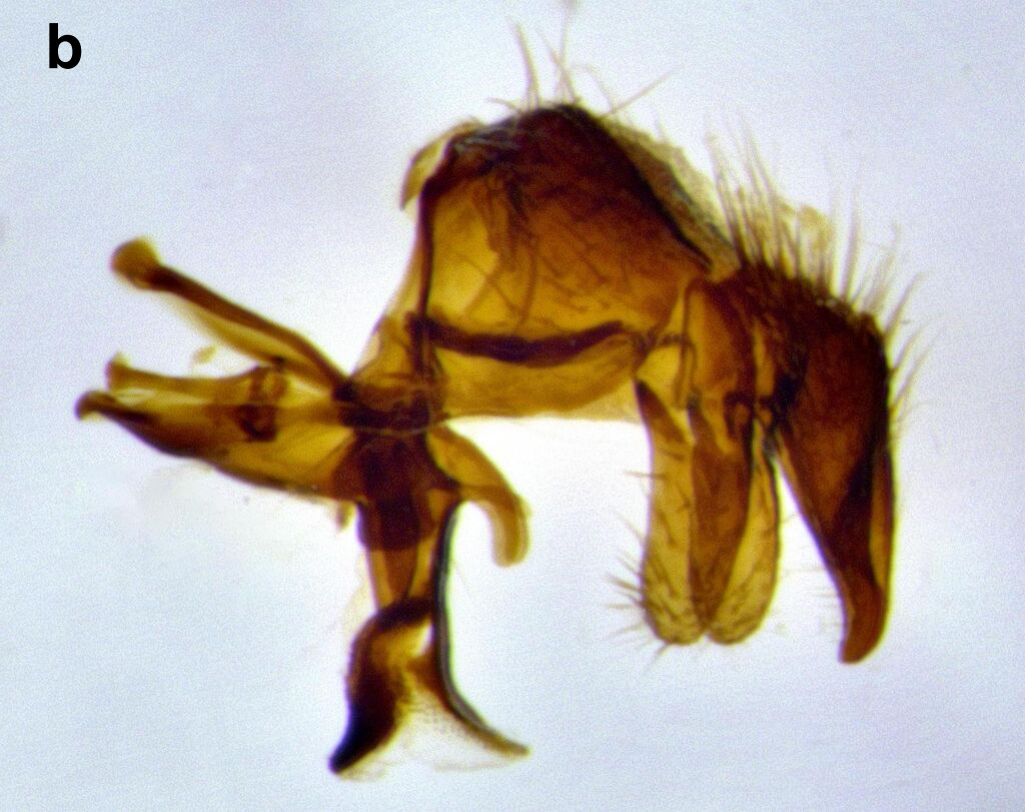
lateral view;

**Figure 11c.**
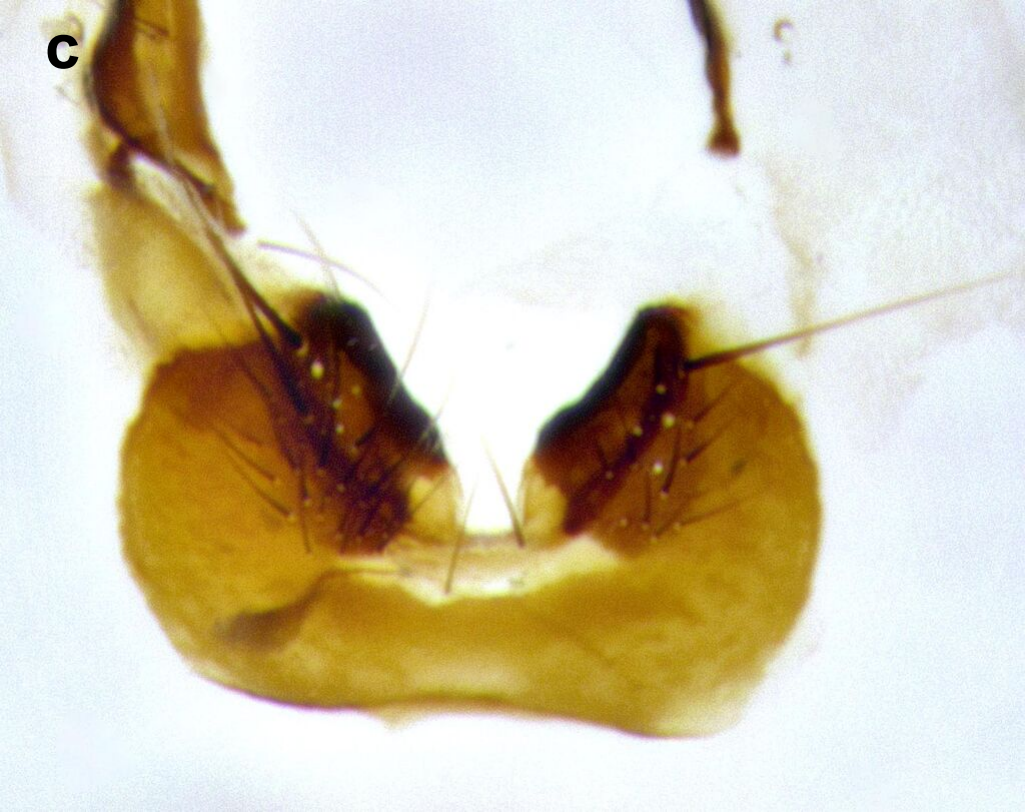
sternite 5;

**Figure 11d.**
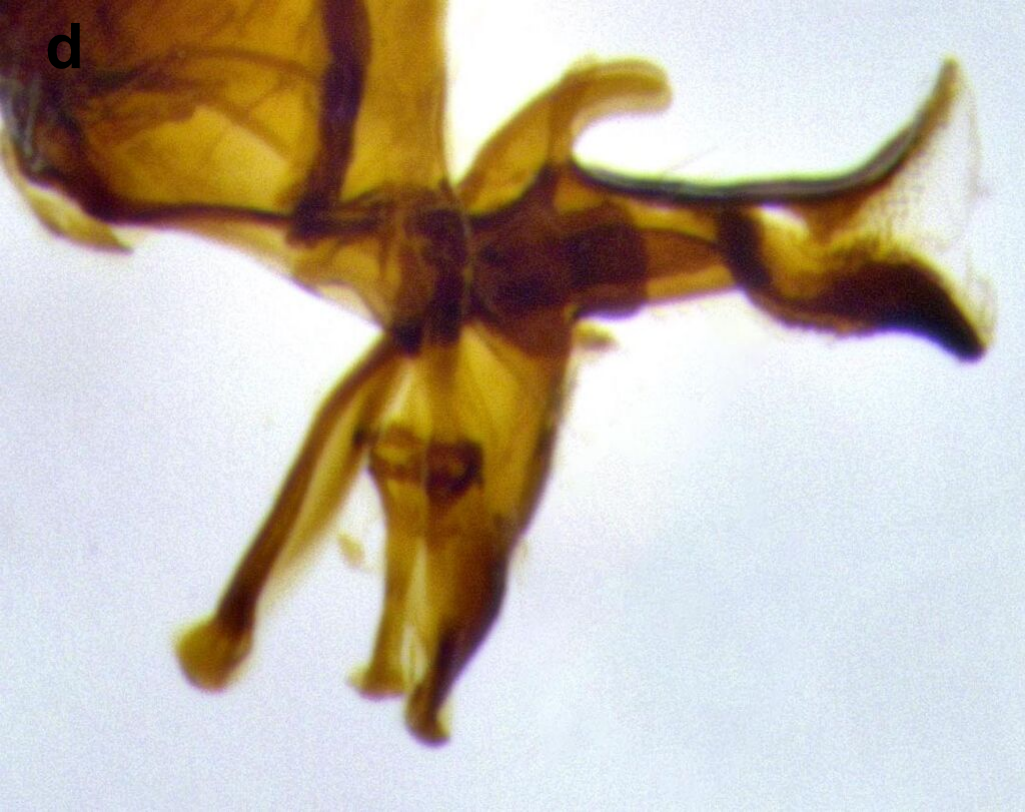
detailed lateral view of distiphallus.

**Figure 12a.**
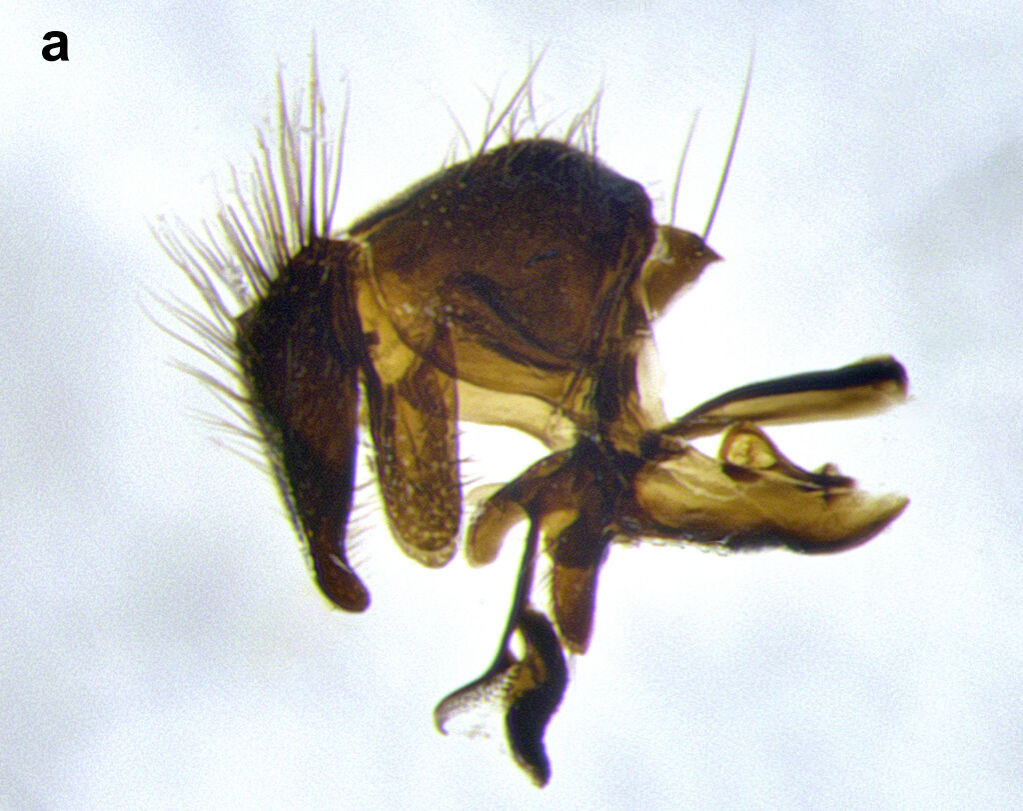
*Santarosamyia woodorum* sp. nov.;

**Figure 12b.**
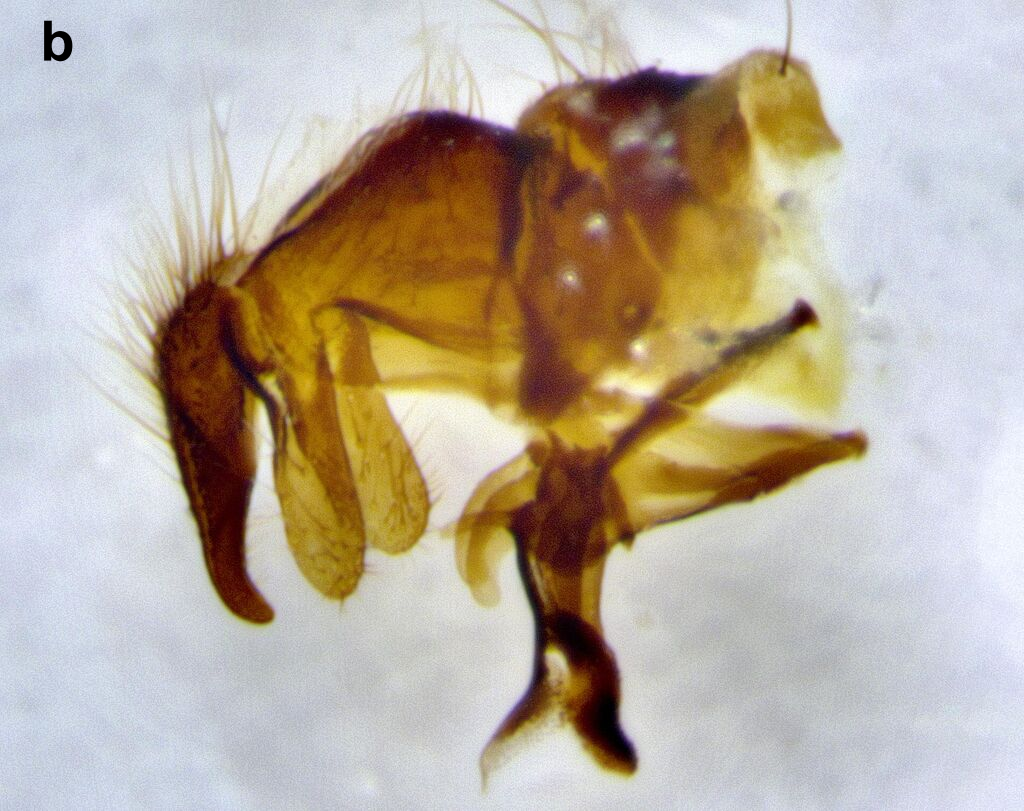
*Santarosamyia erecta* comb. nov.;

**Figure 12c.**
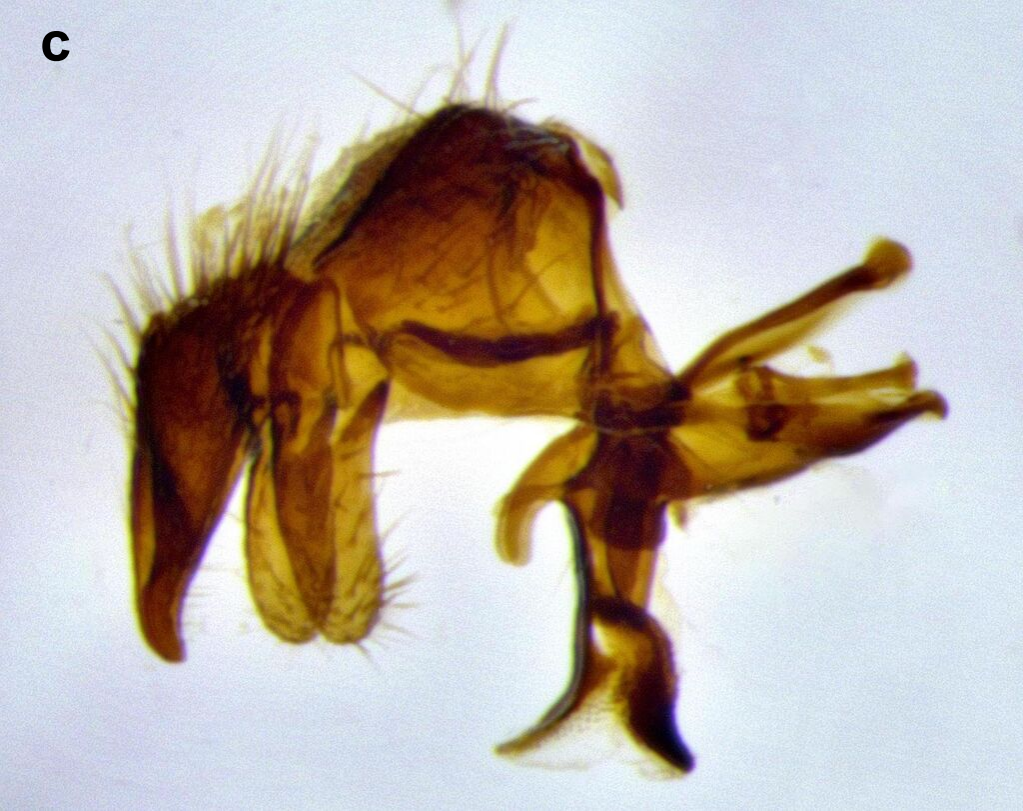
*Santarosamyia unipilum* comb. nov.;

**Figure 12d.**
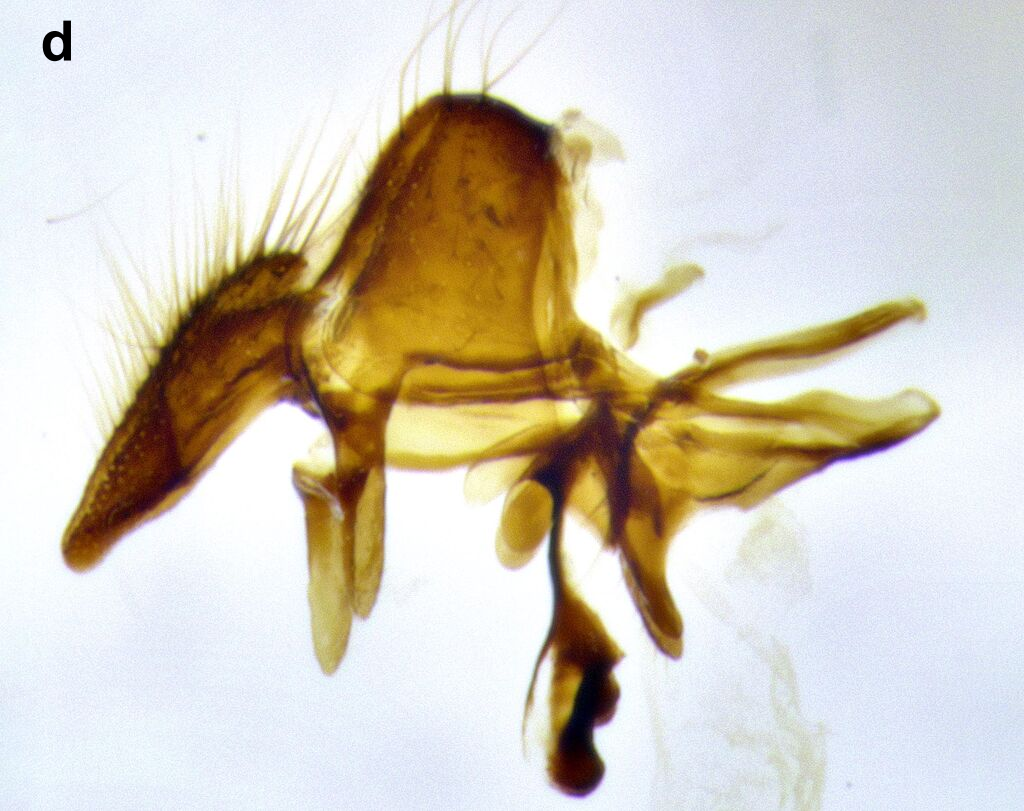
*Nilea innoxia* Robineay-Desvoidy.

## References

[B11226538] Aldrich J. M., Webber R. T. (1924). The North American species of parasitic two-winged flies belonging to the genus *Phorocera* and allied genera. Proceedings of the United States National Museum.

[B11226792] Arnaud P. H. Jr. (1978). A host-parasite catalog of North American Tachinidae (Diptera) Miscellaneous Publication No. 1319.

[B11245073] Bergström Christer (2007). A new species of *Nilea* Robineau-Desvoidy (Diptera, Tachinidae) with notes on the genus and a key to the North European species. Entomologisk Tidskrift.

[B11226819] Cumming J. M., Wood D. M., Kirk-Spriggs A. H., Sinclair B. J. (2017). Manual of Afrotropical Diptera.

[B11226800] Fleming A. J., Wood D. M., Smith M. A., Hallwachs W., Janzen D. H. (2014). Revision of the New World species of *Houghia* Coquillett (Diptera, Tachinidae) reared from caterpillars in Area de Conservación Guanacaste, Costa Rica. Zootaxa.

[B11226885] Fleming A. J., Wood D. M., Smith M. A., Janzen D. H., Hallwachs W. (2015). Nine new species of *Itaplectops* (Diptera: Tachinidae) reared from caterpillars in Area de Conservación Guanacaste, northwestern Costa Rica, with a key to *Itaplectops* species. Biodiversity Data Journal.

[B11226939] Fleming A. J., Wood D. M., Smith M. A., Hallwachs W., Janzen D. H. (2018). Revision of the Mesoamerican species of *Calolydella* Townsend (Diptera: Tachinidae) and description of twenty-three new species reared from caterpillars in Area de Conservación Guanacaste, northwestern Costa Rica. Biodiversity Data Journal.

[B11226917] Fleming A. J., Wood D. M., Smith M. A., Dapkey T., Hallwachs W., Janzen D. H. (2019). Twenty-two new species in the genus *Hyphantrophaga* Townsend (Diptera: Tachinidae) from Area de Conservación Guanacaste, with a key to the species of Mesoamerica. Biodiversity Data Journal.

[B11226928] Fleming A. J., Wood D. M., Smith M. A., Dapkey T., Hallwachs W., Janzen D. H. (2020). A new genus and new species in the tribe Uramyini (Diptera: Tachinidae) from Area de Conservación Guanacaste in northwestern Costa Rica. Biodiversity Data Journal.

[B11226832] Fleming A. J., Woodley N., Smith M. A., Hallwachs W., Janzen D. H. (2023). Revision of *Belvosia* Robineau-Desvoidy (Diptera, Tachinidae) and 33 new species from Area de Conservación Guanacaste in northwestern Costa Rica with a key to known North and Mesoamerican species. Biodiversity Data Journal.

[B11443642] Folmer O, Black M, Hoeh W, Lutz R, Vrijenhoek R (1994). DNA primers for amplification of mitochondrial cytochrome c oxidase subunit I from diverse metazoan invertebrates.. Molecular Marine Biology and Biotechnology.

[B11226773] Guimarães J. H. (1977). Arquivos de Zoologia: Host-parasite and parasite-host catalogue of South American Tachinidae (Diptera).

[B11443652] Hebert Paul D N, Penton Erin H, Burns John M, Janzen Daniel H, Hallwachs Winnie (2004). Ten species in one: DNA barcoding reveals cryptic species in the Neotropical skipper butterfly *Astraptes
fulgerator*.. Proceedings of the National Academy of Sciences of the United States of America.

[B11443662] Hebert Paul D. N., Braukmann Thomas W. A., Prosser Sean W. J., Ratnasingham Sujeevan, deWaard Jeremy R., Ivanova Natalia V., Janzen Daniel H., Hallwachs Winnie, Naik Suresh, Sones Jayme E., Zakharov Evgeny V. (2018). A Sequel to Sanger: amplicon sequencing that scales. BMC Genomics.

[B11443633] Ivanova N. V., DeWaard J. R., Hebert P. D.N. (2006). An inexpensive, automation‐friendly protocol for recovering high‐quality DNA. Molecular Ecology Notes.

[B11227161] Janzen Daniel H., Hallwachs Winnie (2020). Área de Conservación Guanacaste, northwestern Costa Rica: Converting a tropical national park to conservation *via* biodevelopment. Biotropica.

[B11227051] Janzen D. H., Hallwachs W., Blandin Patrick, Burns John M, Cadiou Jean-Marie, Chacon Isidro, Dapkey Tanya, Deans Andrew R, Epstein Marc E, Espinoza Bernardo, Franclemont John G, Haber William A, Hajibabaei Mehrdad, Hall Jason P W, Hebert Paul D N, Gauld Ian D, Harvey Donald J, Hausmann Axel, Kitching Ian J, Lafontaine Don, Landry Jean-François, Lemaire Claude, Miller Jacqueline Y, Miller James S, Miller Lee, Miller Scott E, Montero Jose, Munroe Eugene, Green Suzanne Rab, Ratnasingham Sujeevan, Rawlins John E, Robbins Robert K, Rodriguez Josephine J, Rougerie Rodolphe, Sharkey Michael J, Smith M Alex, Solis M Alma, Sullivan J Bolling, Thiaucourt Paul, Wahl David B, Weller Susan J, Whitfield James B, Willmott Keith R, Wood D Monty, Woodley Norman E, Wilson John J (2009). Integration of DNA barcoding into an ongoing inventory of complex tropical biodiversity.. Molecular Ecology Resources.

[B11227034] Janzen D. H., Hallwachs W. (2011). Joining inventory by parataxonomists with DNA barcoding of a large complex tropical conserved wildland in Northwestern Costa Rica. PLOS One.

[B13238217] Nei Masatoshi, Kumar Sudhir (2023). Molecular Evolution and Phylogenetics.

[B11245122] O'Hara J. E., Henderson J. H., Wood D. M. Preliminary checklist of the Tachinidae (Diptera) of the WorldPreliminary checklist of the Tachinidae (Diptera) of the world. Version 2.1. PDF document, 1039 pp.. http://www.nadsdiptera.org/Tach/WorldTachs/Checklist/Tachchlist_ver2.1.pdf.

[B11227152] Ratnasingham SUJEEVAN, Hebert PAUL D. N. (2007). BOLD: The Barcode of Life Data System (http://www.barcodinglife.org). Molecular Ecology Notes.

[B11443624] Rohland Nadin, Reich David (2012). Cost-effective, high-throughput DNA sequencing libraries for multiplexed target capture. Genome Research.

[B11225720] Seyyedi-Sahebari F., Khaghaninia S., Talebi A. A. (2021). Review of the tribe Eryciini Robineau-Desvoidy (Diptera: Tachinidae: Exoristinae) from Iran, with new records. Journal of Agricultural Science and Technology.

[B11227102] Smith David, Janzen Daniel, Hallwachs Winnie, Smith M. Alex (2012). Hyperparasitoid wasps (Hymenoptera, Trigonalidae) reared from dry forest and rain forest caterpillars of Area de Conservación Guanacaste, Costa Rica. Journal of Hymenoptera Research.

[B11227130] Smith M. Alex, Woodley Norman E., Janzen Daniel H., Hallwachs Winnie, Hebert Paul D. N. (2006). DNA barcodes reveal cryptic host-specificity within the presumed polyphagous members of a genus of parasitoid flies (Diptera: Tachinidae). Proceedings of the National Academy of Sciences.

[B11227120] Smith M. Alex, Wood D. Monty, Janzen Daniel H., Hallwachs Winnie, Hebert Paul D. N. (2007). DNA barcodes affirm that 16 species of apparently generalist tropical parasitoid flies (Diptera, Tachinidae) are not all generalists. Proceedings of the National Academy of Sciences.

[B11227140] Smith M. Alex, Rodriguez Josephine J., Whitfield James B., Deans Andrew R., Janzen Daniel H., Hallwachs Winnie, Hebert Paul D. N. (2008). Extreme diversity of tropical parasitoid wasps exposed by iterative integration of natural history, DNA barcoding, morphology, and collections. Proceedings of the National Academy of Sciences.

[B11227111] Smith M. ALEX, Fernandez‐Triana JOSE, Roughley ROB, Hebert PAUL D. N. (2009). DNA barcode accumulation curves for understudied taxa and areas. Molecular Ecology Resources.

[B13253761] Sterling Mark J., Lees David C., Grundy Dave (2023). Xenotorodor stygioxanthus gen. nov., sp. nov. (Lepidoptera, Lecithoceridae, Torodorinae), described from an established population in Spain with discussion of taxonomic placement. Nota Lepidopterologica.

[B11226783] Stireman John O. (2002). Phylogenetic relationships of tachinid flies in subfamily Exoristinae (Tachinidae: Diptera) based on 28S rDNA and elongation factor‐1α. Systematic Entomology.

[B11226548] Stireman John O., Cerretti Pierfilippo, O'Hara James E., Blaschke Jeremy D., Moulton John K. (2019). Molecular phylogeny and evolution of world Tachinidae (Diptera). Molecular Phylogenetics and Evolution.

[B11226752] Tachi T., Shima H. (2010). Molecular phylogeny of the subfamily Exoristinae (Diptera, Tachinidae), with discussions on the evolutionary history of female oviposition strategy. Systematic Entomology.

[B13238225] Tamura Koichiro, Stecher Glen, Kumar Sudhir (2021). MEGA11: Molecular Evolutionary Genetics Analysis Version 11. Molecular Biology and Evolution.

[B11225712] van Emden F. I. (1954). Diptera: Cyclorrhapha
Calyptrata (I) Sect (a) Tachinidae and Calliphoridae.

[B11226694] Wood D. M., McAlpine J. F., Peterson B. V., Shewell G. E., Teskey H. J., Vockeroth J. R., Wood D. M. (1987). Manual of Nearctic Diptera.

[B11226733] Wood D. M., Zumbado M. A., Brown B. V, Borkent A., Cumming J. M., Wood D. M., Woodley N. E., Zumbado M. A. (2010). Manual of Central American Diptera.

